# VCP interaction with HMGB1 promotes hepatocellular carcinoma progression by activating the PI3K/AKT/mTOR pathway

**DOI:** 10.1186/s12967-022-03416-5

**Published:** 2022-05-13

**Authors:** Zhangya Pu, Dan G. Duda, Yuanyuan Zhu, Siya Pei, Xiaofang Wang, Yan Huang, Panpan Yi, Zebing Huang, Fang Peng, Xingwang Hu, Xuegong Fan

**Affiliations:** 1 Department of Infectious Diseases, Hunan Key Laboratory of Viral Hepatitis, Xiangya Hospital, Central South University, No. 87, Xiangya Road, Kaifu District, Changsha, 410008 Hunan China; 2 NHC Key Laboratory of Cancer Proteomics, Xiangya Hospital, Central South University, No. 87 Xiangya Road, Changsha, 410008 Hunan China; 3 E.L. Steele Laboratories for Tumor Biology, Department of Radiation Oncology, Massachusetts General Hospital, 100 Blossom St, Cox-734, Boston, 02114 USA

**Keywords:** Hepatocellular carcinoma, VCP, HMGB1, PI3K/AKT/mTOR pathway, Tumor progression

## Abstract

**Background:**

Hepatocellular carcinoma (HCC) is the most common pathological type of liver cancer. Valosin-containing protein (VCP) is a member of the AAA-ATPase family associated with multiple molecular functions and involved in tumor metastasis and prognosis. However, the role of VCP in HCC progression is still unclear.

**Methods:**

We examined the expression of VCP in HCC using the RNA sequencing and microarray data from public databases and measured it in clinical samples and cell lines by western blot, and immunohistochemistry (IHC). We also evaluated the correlation between VCP and clinical features. The VCP-interacting proteins were identified by co-immunoprecipitation combined with mass spectrometry (CoIP/MS). The underlying molecular mechanisms were investigated using in vitro and in vivo models of HCC.

**Results:**

We found that VCP expression is significantly increased in tumor tissues and is associated with advanced TNM stages and poorer prognosis in HCC patients. In vitro analyses revealed that VCP overexpression promoted HCC cell proliferation, migration, and invasion via PI3K/AKT/mTOR pathway activation. Conversely, VCP knockdown resulted in the reverse phenotypes. In vivo studies indicated that up-regulated VCP expression accelerated tumor growth in a subcutaneous HCC model. The D1 domain of VCP and A box of HMGB1 were identified as the critical regions for their interaction, and D1 area was required for the tumor-promoting effects induced by VCP expression. VCP enhanced the protein stability of HMGB1 by decreasing its degradation via ubiquitin–proteasome process. Inhibition of HMGB1 markedly attenuated VCP-mediated HCC progression and downstream activation of PI3K/AKT/mTOR signals.

**Conclusion:**

Collectively, these findings demonstrate that VCP is a potential prognostic biomarker in HCC and exhibits oncogenic roles via PI3K/AKT/mTOR pathway activation. HMGB1 played an essential role in VCP-mediated HCC progression, indicating that VCP and HMGB1 are potential therapeutic targets in human HCC.

**Supplementary Information:**

The online version contains supplementary material available at 10.1186/s12967-022-03416-5.

## Introduction

Hepatocellular carcinoma (HCC) is the most prevalent primary liver cancer, accounting for more than 90% of all cases, and has been ranked as the third leading cause of cancer-related death worldwide [1, 2]. Unfortunately, although it has been demonstrated that this is a complex multistep process [3, 4], the molecular mechanisms involved in the tumorigenesis and progression of HCC remain to be fully characterized. Previous studies indicated that several functional genes, such as HBV X protein (HBX) and sperm-associated antigen 5 (SPAG5), are exerting oncogenic activities and intimately associated with HCC progression and prognosis [2, 5, 6]. Therefore, more investigations focused on these functional biomarkers are warranted to better understand the pathogenesis of HCC.

Valosin-containing protein (VCP) is a prominent member of the hexameric AAA family (ATPases associated with various cellular activities) [7, 8]. Structurally, VCP contains two ATPase domains, including the D1 and D2 region, conferring VCP interacts with more than 30 various cellular proteins. It was widely reported that VCP is involved in multiple cellular processes, such as autophagy, endosomal trafficking, and ubiquitin/proteasome-dependent protein degradation [7, 9]. However, despite the significant role of VCP in various biological processes and its abundant substrates for conjunction, the scope and character of its functions in human cells still left a large space for further elucidation. Recently, several studies reported that VCP is overexpressed in certain tumor types and associated with poor prognosis, including HCC, gastric cancer, and non-small cell lung carcinoma (NSCLC). Prior studies were clinical retrospective investigations without further exploration of the molecular mechanisms [10–12]. Asai et al. found that the elevated VCP expression decreased the apoptotic level of osteosarcoma cells, while the metastatic possibility was enhanced. Molecularly, the NF-κB signaling was constitutively activated [13]. Thus, current knowledge concerning the biological function of VCP in the progression of tumors and the involved pathological processes remains limited.

High-mobility group box 1(HMGB1) is an evolutionarily conserved nonhistone chromatin-binding protein [14, 15]. It not only regulates DNA replication and genome stability via acting as a DNA chaperone but also responds to stimuli like inflammation by being released into the cytoplasmic and extracellular environment [16, 17]. HMGB1 is a significant member of the high-mobility molecular family and contained three crucial domains, namely A box (9–79 aa), B box (89–162 aa), and acidic C-terminal tail (186–215 aa), which allowed HMGB1 to interact with various receptors, such as receptors for advanced glycation end products (RAGE), toll-like receptors (TLRs), and other functional molecules like HBX protein [5, 16, 18]. Moreover, HMGB1 has been demonstrated to play a critical role in the carcinogenesis of various cancer types such as esophageal squamous cell carcinoma, lung cancer, and liver tumors [19–21]. Recently, HMGB1 was reported to regulate several cancer-related signal paths, for example, NF-κB signaling and PI3K/AKT/mTOR pathway [15, 22]. However, the precise molecular regulations of HMGB1 and its role in the progression of tumors are not completely characterized.

In this study, we aimed to elucidate the biological function of VCP in HCC progression, including cell proliferation, migration, and invasion. The underlying molecular mechanisms were further explored. Bioinformatic analysis, clinical samples, co-immunoprecipitation combined with tandem mass spectrometry (CoIP/MS), and in vitro and in vivo experimental models were comprehensively performed. Our data revealed that VCP exerted an oncogenic role in the progression of HCC via interaction with HMGB1 and PI3K/AKT/mTOR pathway activation.

## Materials and methods

### Data acquisition and bioinformatic analysis

The RNA sequencing data of the HCC cohort, including 371 HCC and 50 adjacent non-tumorous samples, was downloaded from The Cancer Genome Atlas Program (TCGA) database (https://portal.gdc.cancer.gov/). The HCC datasets, including GSE121248 (70 HCC and 37 normal samples), GSE136247 (39 HCC and 30 normal samples), GSE41804 (20 HCC and 20 normal samples), and GSE14520 (247 HCC and 241 normal samples) were screened from the Gene Expression Omnibus (GEO) database (https://www.ncbi.nlm.nih.gov/geo/). Concerning the HCC cohort from TCGA database, the GEPIA tool (http://gepia.cancer-pku.cn/) was operated to analyze the correlation of VCP expression and survival probability following the criteria of the best cut-off algorithm based on VCP transcriptional level in patients. Meanwhile, the UALCAN tool (http://ualcan.path.uab.edu/) was performed to obtain all candidate genes co-expressed with VCP according to Pearson’s correlation coefficient.

### Gene set enrichment analysis

Gene set enrichment analysis (GSEA) is a computational algorithm utilized to assess whether a previously defined gene set indicates a statistical significance and concurrent difference between two biological phenotypes [23]. The Kyoto Encyclopedia of Genes and Genomes (KEGG) gene sets were retrieved from the Molecular Signatures Database (MSigDB, version 7.5.1). HCC patients from TCGA database were divided into the high and low expressed groups according to the median transcriptional value of VCP and HMGB1, respectively. The normalized enrichment score (NES) and false discovery rate (FDR) were determined by GSEA software (version 4.1.0). FDR value < 0.05 was thought of as statistical significance.

### Function annotation and protein–protein interaction network

Gene ontology (GO) analysis, including biological process (BP), cell components (CC), and molecular function (MF), and pathway enrichment including KEGG and Reactome panels, were performed using the Metascape online tool (https://metascape.org/). The protein–protein interaction (PPI) network was constructed in the STRING database (https://string-db.org/) and visualized via Cytoscape software (version 3.8.2). The hub genes were identified using the Cytohubba plug-in.

### Cell culture and transfection

Human HCC cell lines of Huh7 and MHCC-LM3 cells, as well as 293T cells, were purchased from the American Type Culture Collection (ATCC, Manassas, VA, USA). The immortalized hepatocyte cell line L02 was obtained from the China Center for Type Culture Collection (CCTCC, China). Huh7, MHCC-LM3, and 293T cells were cultured in Dulbecco’s modified Eagle’s medium (DMEM, Gibco, USA), and L02 cells were maintained in RPMI medium (Gibco). All were supplemented with 10% fetal bovine serum (FBS, Gibco, USA), penicillin (100 U/ml), and streptomycin (100 μg/ml), and cultured in a humidified incubator with 5% CO_2_ at 37 °C.

The stable cell line was constructed with lentivirus transduction using Lipofectamine 2000 (Invitrogen, USA) according to the manufacturer’s instructions. Huh7 cells were transfected by PLKO.1-puro-VCPshRNA (VCP-sh1 and VCP-sh2) or PLKO.1-puro-nonspecific shRNA (as control), MHCC-LM3 cells were treated with PLV-EGFP-puro-Myc-VCP or PLV-EGFP-puro plasmid (as control). Then, HCC cells with successful lentivirus transfection were screened by puromycin for 10 to 14 days. Small interfering RNA (siRNA) and various plasmids were transfected into cells using Lipofectamine 2000. HMGB1-siRNA target sequences (5′ to 3′) #1: CCCGTTATGAAAGAGAAATTT. #2: GGAGGAAGATGAAGAAGATTT. VCP-siRNA target sequences (5′ to 3′) #1: GAATAGAGTTGTTCGGAAT. #2: GGCCAAAGCCATTGCTAAT.

### Western blot and antibodies

Whole-cell lysates were collected using the protein lysis buffer containing proteinase and phosphatase inhibitors. The BCA assay was operated to measure the protein concentration. The protein was separated by 12% or 10% sodium dodecyl sulfate–polyacrylamide gel electrophoresis (SDS-PAGE) gels. The gel was then transferred to polyvinylidene difluoride (PVDF) membranes (0.25 μm, Millipore, Billerica, MA, USA), and blocked with 5% fat-free dry milk resolved in TBST buffer. The membranes were then washed three to five times (5 min/time) and incubated with the indicated primary antibodies including VCP (2648, CST, USA), HMGB1 (ab18256, Abcam, USA), E-cadherin (20874-1-AP, Proteintech, China), β-catenin (51067-2-AP, Proteintech, China), Snail (sc-271977, Santa Cruz, USA), Twist2 (66544-1-Ig, Proteintech, China), PI3K (4249, CST, USA), AKT (10176-2-AP, Proteintech, China), pAKT (66005-1-Ig, CST, USA), mTOR (20657-1-AP, Proteintech, China), pmTOR (5536, CST, USA), ub (sc-8017, Santa Cruz, USA), GAPDH (60004-1-Ig, Proteintech, China), Myc-tag (60003-2-Ig, Proteintech, China), His-tag (12698, CST, USA), and Flag-tag (66008-3-Ig, Proteintech, China). The bound antibodies were examined using enhanced chemiluminescent reagents (34577, Thermo Fisher, USA) after incubation with horseradish peroxidase (HRP)-conjugated secondary antibodies (FSM0075 and FSM0056, Fushen, China). The relative protein quantification was analyzed using ImageJ software.

### Quantitative real-time PCR (qPCR)

Total RNA was extracted using TRIzol reagent (15596018, Invitrogen, USA) following the manufacturer’s instructions. Reverse transcription (PrimeScript RT reagent Kit, RR047A, Takara, Japan) and SYBR (1725124, Bio-Rad, USA) green-based real-time PCR were performed. GAPDH expression was regarded as endogenous control, and the value of 2^−ΔΔCT^ was adopted to determine the relative gene transcriptional expression. The qPCR primers included VCP forward: 5′-CTGGAGCCGATTCAAAAGGTG-3′, reverse: 5′-ACACTGTGTCACCTCGGAAC-3′. HMGB1 forward: 5′-GCGAAGAAACTGGGAGAGATGTG-3′, and reverse: 5′-GCATCAGGCTTTCCTTTAGCTCG-3′.

### Immunohistochemistry (IHC) 

Forty-four HCC samples and adjacent non-tumorous tissues from HCC patients who underwent surgical resection were collected at Xiangya Hospital, Central South University (CSU), Changsha, China, between March 2017 and March 2020. Written informed consent was obtained from all patients. This project was approved by the Medical Ethics Committee of the Xiangya Hospital, CSU. Immunohistochemistry was performed as described previously [24]. The VCP expressed level was evaluated using Image J software, and semi-quantitatively scored as highly positive (4), moderately positive (3), low positive (2), and negative/undetectable (1) staining.

### Mass spectrometry (MS)

The LC-ESL-LTQ-Orbitrap-MS method was used to identify VCP-interacting proteins as described in a previous study [25]. Briefly, the protein bands were cut from the gel and transferred to 100 mM NH_4_HCO_3_ with 50% acetonitrile for excising and destaining. Subsequently, the proteins were reduced, alkylated, and dried in a vacuum centrifuge. The gel pieces harboring proteins were incubated in digestion solution at 37 °C for 18–24 h. The tryptic peptide mixture was purified with a ZipTipC18 microcolumn (ZTC18S096, Millipore, Germany) and subjected to separation on a Pep Map C18 trap column (75 μm, 15 cm) with column flow rates of 200 nL/min. MS/MS analysis of the seven strongest ions in the LTQ was conducted using MS and MS spectra. Next, Xcalibur and Proteome Discoverer software were used to analyze the MS data.

### Immunofluorescence (IF) 

Cells were cultured in twelve-well plates with coverage of glass coverslips up to 30–40% confluency. The cells were washed and fixed with 4% paraformaldehyde before blocking with 5% BSA containing 0.1% Triton-100. Then, the cells were incubated with mouse HMGB1(SAB1403925, Sigma, USA) and rabbit VCP (2648, CST, USA) primary antibodies at 4 °C overnight. Next, the cells were stained with Alexa Fluor 488-conjugated anti-mouse IgG (A-21202, Thermo Fisher, USA) or Alexa Fluor 594-conjugated anti-rabbit IgG (A-11012, Thermo Fisher, USA) for 1 h at room temperature. After intermediate washes, fluorescent signals were detected under a confocal microscope (Olympus, Tokyo, Japan).

### Immunoprecipitation (IP)

Cells were lysed in IP specific RIPA buffer (P0013D, Beyotime, China) and centrifuged for 20 min at 12,000*g*. The lysed samples were then incubated with protein A/G agarose beads (sc-2003, Santa Cruz, USA) for 2 h at 4 °C to prevent non-specific binding and spin. Subsequently, the proteins were incubated with specific antibodies or the same species of IgG (1 μg antibody: 1 mg cellular protein) overnight at 4 °C. Then, protein A/G agarose (20–80 μL) was added again for 3 h incubation, followed by extensive washing with RIPA buffer. Finally, the precipitated proteins were eluted by boiling in the 2 × SDS sample buffer and subjected to SDS-PAGE.

### GST pull-down

GST Sefinose Resin (C600031, Sangon Biotech, China) was used to purify the GST-tagged VCP protein complex. The purified proteins were examined using Coomassie blue staining. The lysate from MHCC-LM3 cells was incubated with GST-tagged proteins at 4 °C overnight with gentle rotation. The precipitated pellets were washed to elute resins and bound proteins were analyzed by SDS-PAGE.

### Cell proliferation and apoptosis assays

For Cell Counting Kit-8 (C0038, Beyotime, China), cells were seeded at a density of 1000 cells/well into 96-well plates and cultured for 24 h, 10 μL CCK8 was added to each well and incubated for 2 h. The absorbance at 450 nm was measured using a PerkinElmer spectrophotometer. For the EdU Cell Proliferation Kit (C0078S, Beyotime, China), appropriate cells were seeded into 24-well plates and incubated for 24–36 h. EdU (20 μM) was added to each well and incubated for 2 h. Then, the cells were fixed with 4% paraformaldehyde and incubated with phosphate-buffered saline (PBS) containing 0.3% Triton X-100. Next, the cells were incubated with 0.5 mL Click Additive solution for 30 min followed by the nuclei stained with DAPI (1:1000) for 10 min in the dark. The fluorescence was examined under a fluorescence microscope.

For the apoptosis assay, the cells were digested with trypsin and washed twice with sterile PBS. Next, the cells were centrifuged for 5 min at 1000*g*. Then, cells were incubated with Annexin V labeled with PE and 7-Amino-Actinomycin (7AAD) for 15 min (559763, BD Biosciences, USA) at room temperature in the dark. Apoptotic cells were screened by flow cytometric analysis.

### Wound-healing, migration, and invasion assay

For the wound-healing assay, 1 × 10 ^6^ cells were seeded into six-well plates. When the cells reached 90% confluence, the cell layer was scratched with a 10 μL sterile plastic tip and cultured for 72 h in serum-free medium. Images were taken under a microscope at the indicated time point to evaluate the healing rate of gap closure.

For the transwell system, 5 × 10^4^ cells were plated in the upper compartment of a transwell chamber (8 μm size, 3422, Corning, USA) in a serum-free medium. The upper chamber was coated with 10% Matrigel (356234, Corning, USA) when for invasion assay. The lower chamber was filled with fresh medium containing 10% FBS. After incubation of 24–48 h, the cells on the lower membrane were fixed with 4% paraformaldehyde and stained with crystal violet (C0121, Beyotime, China). Matrigel-invading or migratory cells were counted under a microscope.

### Animal model

Male BALB/c nude mice aged 4–5 weeks were purchased from HFK Bioscience (Beijing, China) and randomized into various groups, with 6–7 mice in each group. One million MHCC-LM3 cells with stable overexpressing VCP or 2.5 million Huh7 cells with stable silencing VCP (suspended in 0.1 mL PBS) were implanted subcutaneously under the right armpit of nude mice. Body weight and tumor size were measured every three days. The mice were sacrificed three weeks later or when they are moribund. Tumor volume (mm^3^) was calculated using the longest diameter × (shortest diameter)^2^ × 0.5. This experiment was performed in compliance with the requirements of the Department of Laboratory Animals, Xiangya Hospital, CSU, China.

### Statistical analysis

For quantitative variables, a student’s t-test with one-tailed was used to compare the difference between two groups. When more than two groups are included in the experiments, the one-way ANOVA with a Brown-Forsythe test was accepted for multiple comparisons. The chi-square test was conducted for the qualitative variables. Kaplan–Meier method with a Log-rank test was operated to compare the survival distributions. All analyses were performed in GraphPad Prism software (version 8.0) and the results are presented as the mean ± SD or mean ± SEM. Differences were considered statistical significance at a P value < 0.05. Experimental data were obtained from three independent experiments unless otherwise presented. All *P < 0.05, **P < 0.01, ***P < 0.001, ****P < 0.0001, and ns: no significance.

## Results

### VCP expression is increased and correlated with aggressive disease and poor prognosis, and promotes HCC growth in vivo

**Fig.1:**
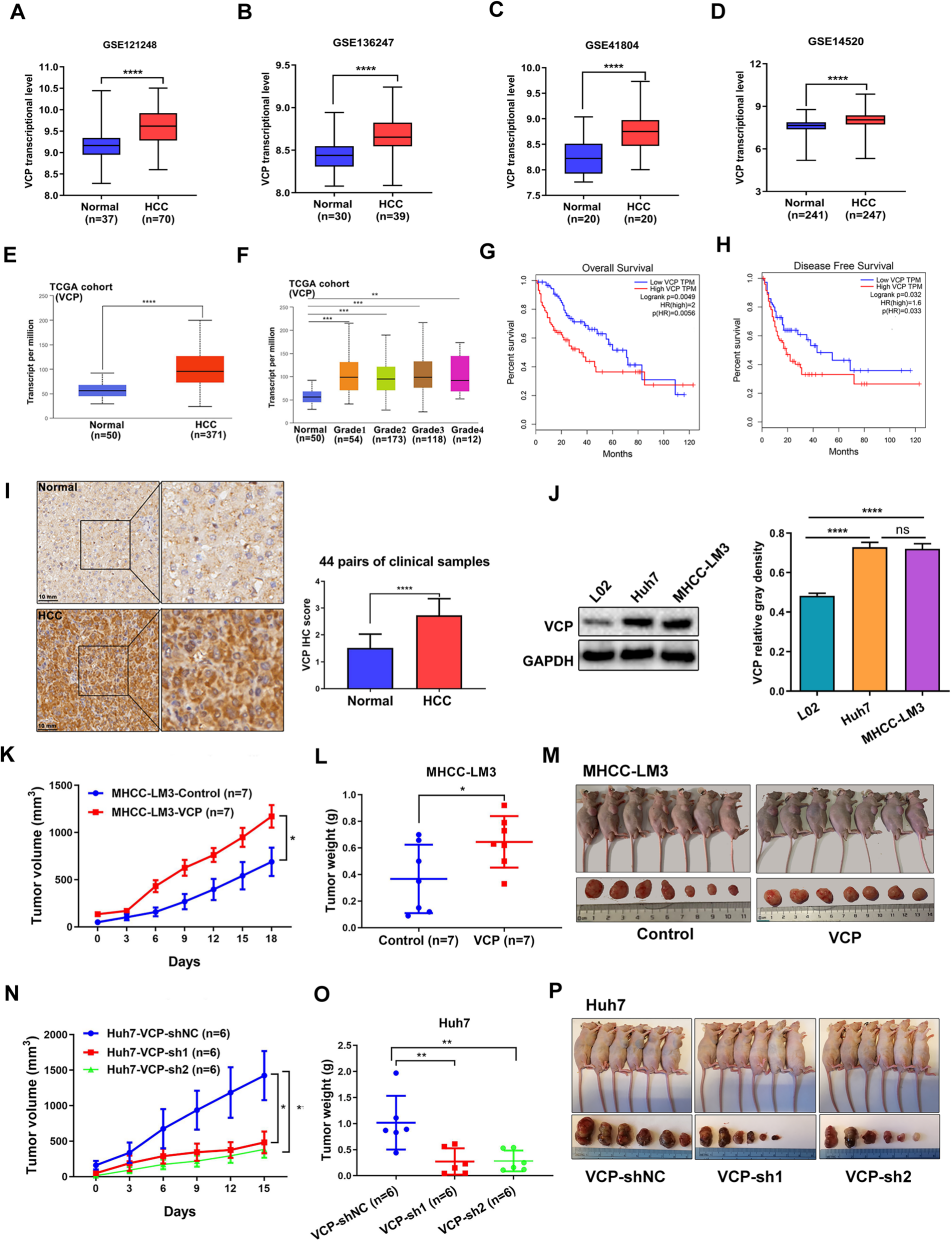
Elevated VCP expression is associated with poor prognosis and promotes HCC growth in vivo. **A**–**D** The mRNA expression of VCP was elevated in HCC samples compared with nontumorous in GEO datasets including GSE121248, GSE136247, GSE41804, and GSE14520. **E**, **F** The HCC cohort from TCGA database presented the increase of VCP in tumor samples and various disease grades. **G**, **H** The clinical significance of VCP expression in overall and disease-free survivals in HCC patients was evaluated in the TCGA cohort. **I** The expression of VCP in HCC and normal tissues from 44 pairs of clinical samples was detected by IHC. The representative images and the statistical result of IHC scores were displayed. **J** The protein expression of VCP in human HCC cell lines (Huh7 and MHCC-LM3) and immortalized hepatic cell line (L02) was determined by western blot. The statistical analysis was further analyzed. **K**, **N** The nude mice were injected with MHCC-LM3 cells with stable VCP overexpression and Huh7 cells with stable VCP depletion, respectively, in the right flank followed by monitoring growth for three weeks. The tumor volumes were measured every 3 days. **L**, **O** The differences in tumor weight in various groups when mice were sacrificed. **M**, **P** The representative morphology of tumors in nude mice. TCGA: The Cancer Genome Atlas. GEO: Gene Expression Omnibus. IHC: immunohistochemistry. All *P < 0.05, **P < 0.01, ***P < 0.001, and ****P < 0.0001

The expression of VCP was first evaluated using public cancer databases. Data analysis using TCGA database showed that VCP is elevated in several tumor types, such as breast cancer, colorectal cancer, and HCC (Additional file 1: Fig. S1A). Data analysis using the GEO database showed that VCP expression was significantly increased in tumor samples compared to the corresponding normal tissue samples (Fig. 1A–D). Further analysis of the difference in VCP expression between tumor and adjacent normal tissues was conducted in the HCC cohort from TCGA, and consistent results were obtained (Fig. 1E, F; Additional file 1: Fig. S1B). In addition, a poorer prognosis, including shorter overall survival (OS) and disease-free survival (DFS), was observed in HCC patients with a higher VCP expression versus those with lower expression levels (Fig. 1G, H). The correlation between VCP expression and clinicopathological factors in HCC patients was further analyzed in GSE14520 dataset. The results indicated that the up-regulated VCP expression was significantly associated with the advanced tumor-node-metastasis (TNM) stage (P-value = 0.03) (Additional file 2: Table S1).

Furthermore, 44 pairs of HCC and adjacent normal liver samples were collected from Xiangya Hospital. The results of IHC staining indicated the markedly increased VCP expression in tumor tissues compared to the adjacent normal tissues (Fig. 1I). Consistently, the VCP protein expression level in HCC cells was higher than that in immortalized normal liver cells (Fig. 1J). Additionally, we used subcutaneous xenograft mice models to determine whether VCP facilitates HCC growth in vivo. The results revealed that tumor growth rate was significantly accelerated in the group overexpressing VCP. Reversely, the growth ability was inhibited in the group of VCP depletion (Fig. 1K–P). Overall, these data suggest that the overexpression of VCP may serve as a poor prognostic factor in HCC and promotes tumor progression.

### VCP promotes HCC cell proliferation

**Fig. 2:**
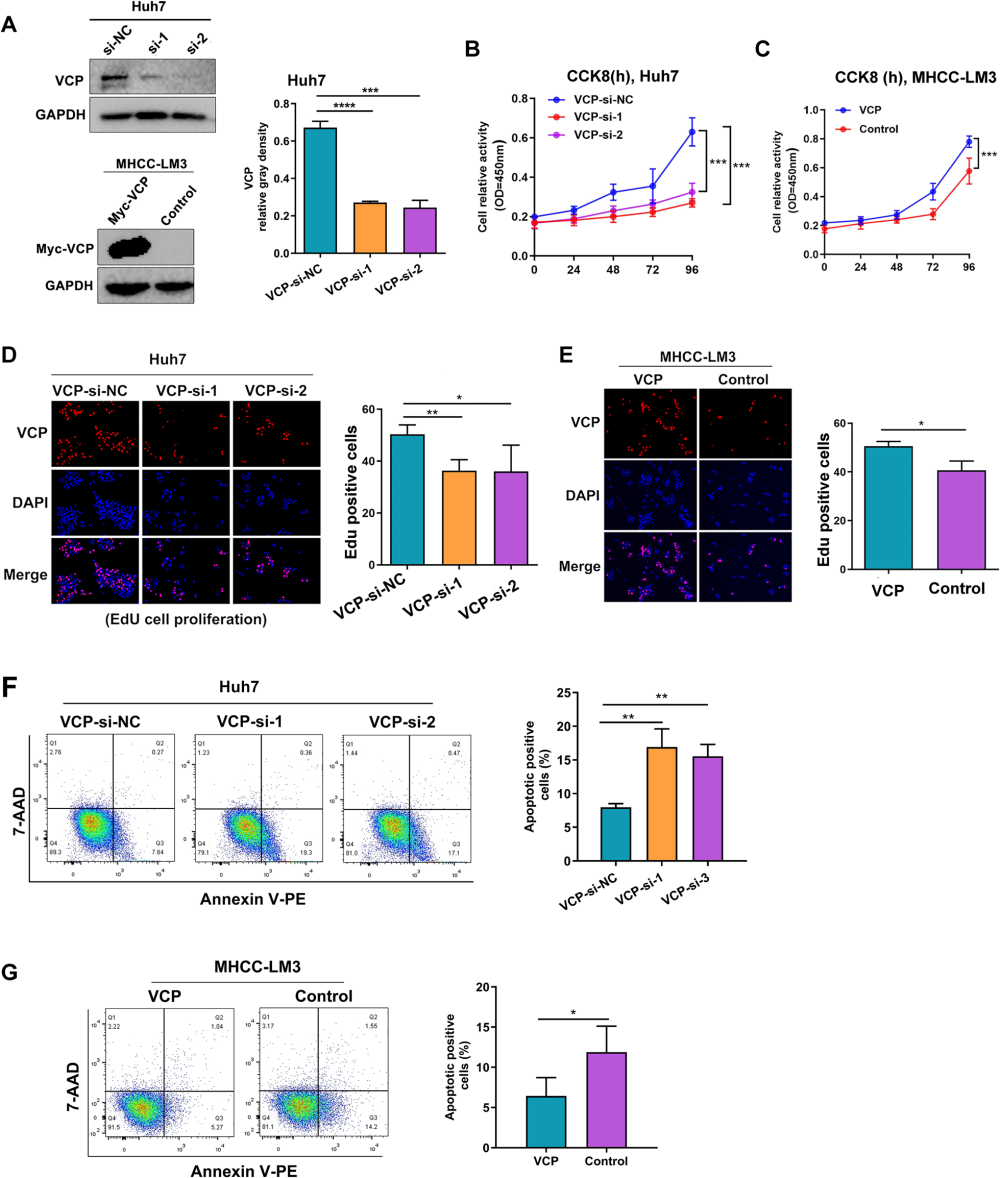
The effect of VCP on HCC cell proliferation and apoptosis in vitro. **A** The efficacy verification of VCP-siRNA and exogenous VCP transfection in HCC cells. **B**, **C** CCK8 assay was performed to measure the cell viability. **D**, **E** The EdU experiment was also used to detect cell ability of proliferation induced by different expressed levels of VCP. **F**, **G** The apoptotic level of HCC cells was determined by flow cytometry. All *P < 0.05, **P < 0.01, and ***P < 0.001

We next examined the biological functions of VCP in HCC. Specific siRNAs targeting VCP were transfected into Huh7 cells and MHCC-LM3 cells were treated with exogenous VCP (Fig. 2A). The CCK8 assay targeting the cytoplasm and the EdU assay targeting the nucleus were conducted to detect cell proliferative ability. The results found that knockdown of VCP markedly inhibited the growth ability of HCC cells, as indicated by both assays (Fig. 2B–E). Flow cytometry was performed to identify the potential mechanism by which VCP promotes cell proliferation in HCC. The results indicated that the inhibition of VCP induced an obvious increase in apoptotic levels in Huh7 cells. Conversely, overexpression of VCP significantly decreased the fraction of apoptotic cells in MHCC-LM3 cells (Fig. 2F, G).

### VCP facilitates HCC cell migration and invasion

**Fig. 3:**
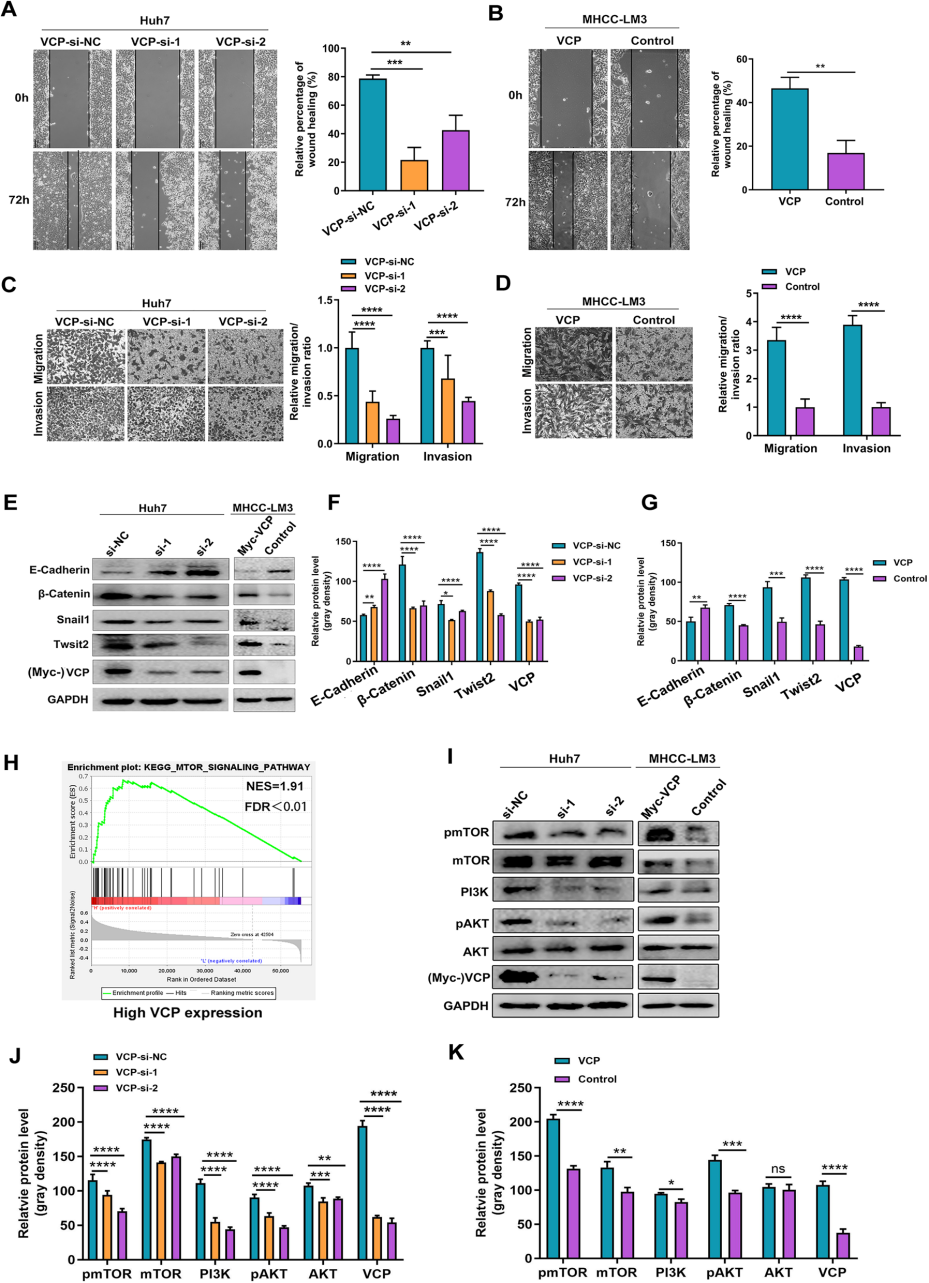
VCP expression promotes migration and invasion in HCC in vitro. **A**, **B** the expression of VCP was silenced in Huh7 cells treated with siRNAs and exogenously overexpressed in MHCC-LM3 cells. Wound-healing assay demonstrated cell movement capacity. **C**, **D** The transwell system was utilized to detect the migration and invasion abilities of HCC cells. The representative images and the corresponding statistical results were shown. **E**–**G** The protein expression of EMT-related markers was determined by western blot and the relative protein density was analyzed. **H** The GSEA analysis in the HCC cohort from TCGA database based on the VCP transcriptional expression showed the up-regulated PI3K/AKT/mTOR pathway. The median value of VCP transcriptional level was regarded as the cut-off to divide HCC patients into high- or low-VCP expression groups. **I**–**K** Proteins extracted from Huh7 and MHCC-LM3 cells in various groups were subjected to western blot to detect the markers within the mTOR pathway. Meanwhile, the relative protein gray density was summarized. *EMT* epithelial-mesenchymal transition, *GSEA* gene set enrichment analysis, *FDR* false discovery rate, *NES* normalized enrichment score, *EMT* epithelial-mesenchymal transformation, *ns* no significance. All *P < 0.05, **P < 0.01, ***P < 0.001, ****P < 0.0001

The correlation analysis of clinicopathologic traits and VCP expression showed that the elevated expression of VCP is associated with poor prognosis in HCC patients. The influence of VCP on the migratory and invasive abilities of HCC cells was further assessed. The wound-healing assay revealed that Huh7 cells transfected with VCP siRNA exhibited lower migratory ability than cells transfected with non-specific siRNA. A similar result was observed in MHCC-LM3 cells in which overexpressing VCP accelerated the migratory speed (Fig. 3A, B). Consistently, the transwell assay also revealed that VCP depletion decreased the ratio of migration and invasion in Huh7 cells, whereas overexpressing VCP enhanced this process in MHCC-LM3 cells (Fig. 3C, D). These data indicated that VCP may correlate with tumor metastasis. The markers related to epithelial-mesenchymal transition (EMT) were further examined by western blot. It was noticed that VCP overexpression promoted the expressed level of β-catenin, Snail1, and Twist2, but the phenomenon was attenuated by VCP knockdown in HCC cells. In contrast, the expression level of E-cadherin was downregulated when overexpressing VCP (Fig. 3E–G).

To identify possible tumor-related pathways involved in the process of VCP facilitating the migration and invasion in HCC cells, GSEA analysis was performed in the HCC cohort from TCGA based on the transcriptional expression of VCP. The median value was regarded as the cut-off to classify patients into high- and low-VCP expression groups. The results indicated the up-regulation of mTOR signaling pathway in the group with high VCP expression, which is known to associate with tumor progression (Fig. 3H) [2, 26]. Several critical biomarkers within the mTOR pathway were determined by western blot in HCC cells with silencing or exogenously overexpressing VCP. The results demonstrated that the expression of PI3K was upregulated by increasing VCP expression, and the phosphorylated levels of AKT and mTOR were also elevated. These effects were attenuated when VCP expression was inhibited (Fig. 3I–K).

### Identification of VCP-interacting proteins

It was reported that VCP belongs to the AAA-ATPase family, which contains two critical ATPase domains, namely the D1 and D2 areas, empowering its ability to bind to a variety of substrates. Sequentially, it exhibits diverse functions in multiple biological processes, such as autophagy and proteasome-dependent protein degradation [8]. In the current study, aiming to clarify the potential regulatory mechanism of VCP promoting HCC cell migration and invasion, the CoIP/MS technology was adopted to identify VCP-interacting proteins in MHCC-LM3 cells, and a total of 330 proteins were identified (Additional file 3: Table S2). The overall profile of the biological functions was analyzed for the candidate proteins via GO and pathway enrichment. The results indicated that VCP is involved in several biological processes, such as protein binding and various diseases, including HCC (Additional file 4: Figure S2).

**Fig. 4:**
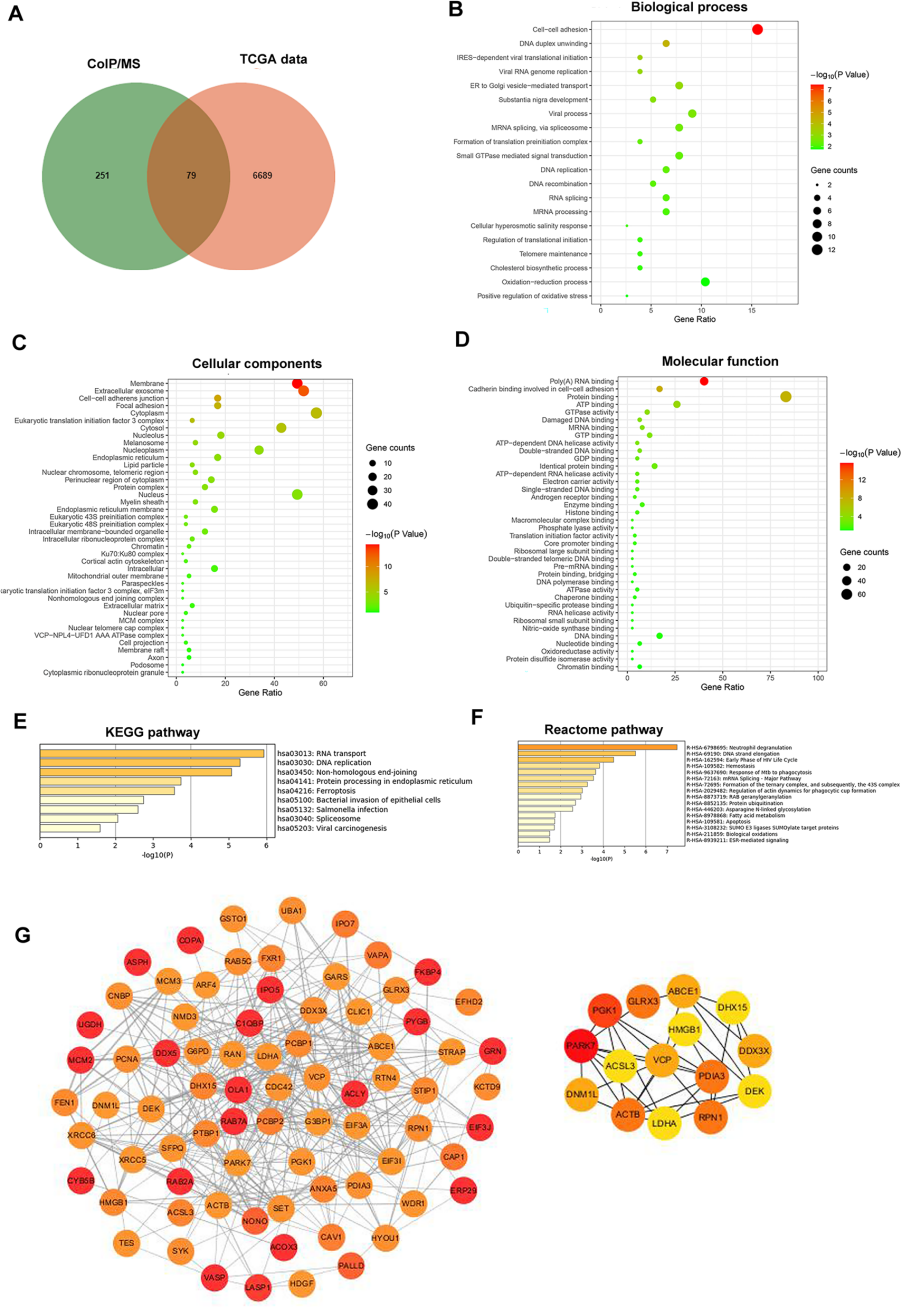
The GO and pathway enrichment, and protein to protein (PPI) network analysis of VCP-interacting proteins. **A** The Venn diagram showed 79 VCP-interacting proteins obtained by overlapping the candidates of CoIP/MS and the profile of VCP co-expressed genes in the HCC cohort from TCGA database. **B**–**D** The GO enriched terms of top 20 biological processes, cell components, and molecular function, respectively. **E**, **F** The enriched pathways through KEGG and Reactome database, separately. **G** The PPI network of 79 VCP-interacting proteins was analyzed in STRING database and further visualized by Cytoscape software (version 3.8.2). A total of 15 Hub genes were determined by CytoHubba plug-in. The colors of nodes from red to yellow indicated the overall combined scores of each molecule interacting with others from high to low. *GO* gene ontology, *KEGG* Kyoto Encyclopedia of Genes and Genomes

**Fig. 5:**
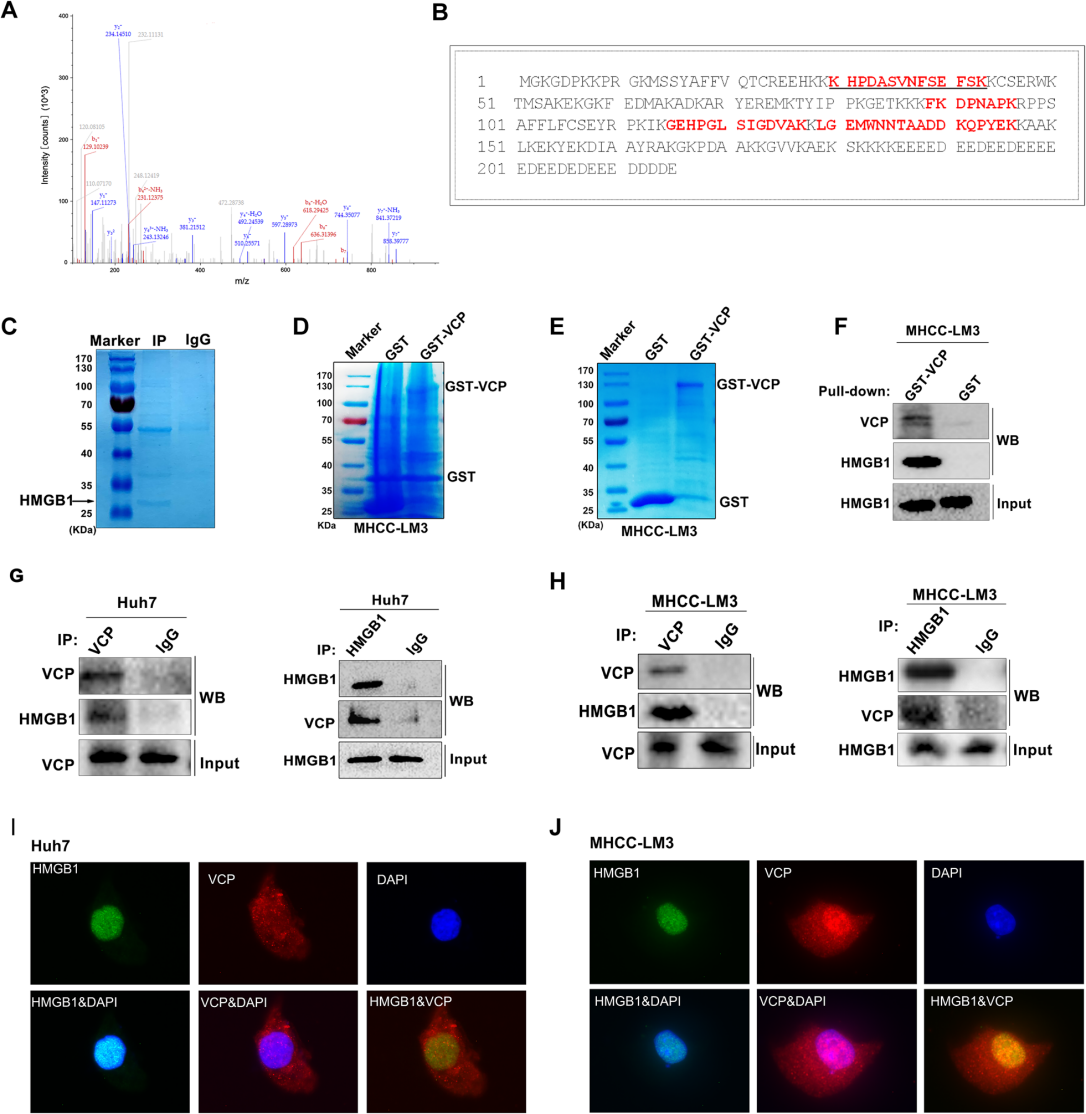
VCP interacts with HMGB1 in HCC cells. **A** The representative MS/MS spectrum of HMGB1 and the amino acid sequence was identified as KHPDASVNFSEFSK from mass differences in the y and b fragment ions series. **B** The protein sequence of HMGB1, the matched peptides are labeled in red bold letters. **C** MHCC-LM3 cells were treated with exogenous VCP and immunoprecipitation was performed. The HMGB1 protein shown by the arrow was observed by Coomassie staining after running SDS-PAGE. **D**–**F** GST pull-down assay indicated that VCP could be fused (**D**) and purified (**E**) in vitro, and the purified GST-tagged VCP pulls down HMGB1 in MHCC-LM3 cells (**F**). **G**, **H** The CoIP was operated to validate the VCP-HMGB1 interaction in Huh7 and MHCC-LM3 cells. **I**, **J** The colocalization of VCP and HMGB1 in HCC cells was observed by confocal microscopy. *SDS-PAGE* sodium dodecyl sulfate–polyacrylamide gel electrophoresis, *MS/MS* tandem mass spectrometry, *CoIP* co-immunoprecipitation

Meanwhile, a total of 6768 potential VCP co-expressed genes were retrieved in the HCC cohort from TCGA database according to Pearson's correlation coefficient. Finally, 79 VCP-interacting proteins were obtained after overlapping the protein candidates identified in CoIP/MS and TCGA (Fig. 4A). GO analysis showed that the proteins exhibited significant roles in diverse BPs, CCs, and MFs, including protein complex, cell-to-cell adhesion, ATP binding, and ubiquitin-specific protease binding (Fig. 4B–D; Additional file 5:Table S3). The KEGG and Reactome pathway enrichment indicated that diversified signal panels were enriched, such as protein processing in the endoplasmic reticulum and protein ubiquitination (Fig. 4E, F). The PPI network of VCP and its interacting proteins was constructed in the STRING database, and the hub genes that have the highest possibility of binding with VCP were further identified (Fig. 4G). HMGB1 was part of the protein complexes after VCP immunoprecipitation in MHCC-LM3 cells as well as in the hub gene network (Figs. 4G, 5C). Then, HMGB1 was selected for further experimental investigation. The MS/MS spectrum of HMGB1 is shown, and the identified amino acid sequence that interacted with VCP is labeled in red color (Fig. 5A, B).

### VCP interacts with HMGB1 in HCC cells

We next sought to validate the interaction between VCP and HMGB1 in HCC cells. First, the GST-tagged VCP fusion protein was successfully purified using GST agarose beads (Fig. 5D, E). The GST pull-down experiment was conducted by incubating GST-tagged VCP with whole lysates from MHCC-LM3 cells. HMGB1 was detected in the GST-tagged VCP pulled down samples but not in the GST-tagged control group (Fig. 5F). Consistently, the CoIP assay demonstrated that endogenous VCP and HMGB1 could be precipitated with each other in HCC cells as well (Fig. 5G, H). Moreover, the confocal assay was performed to show the co-localization of VCP and HMGB1 in HCC cells in vivo (Fig. 5I, J).

**Fig. 6:**
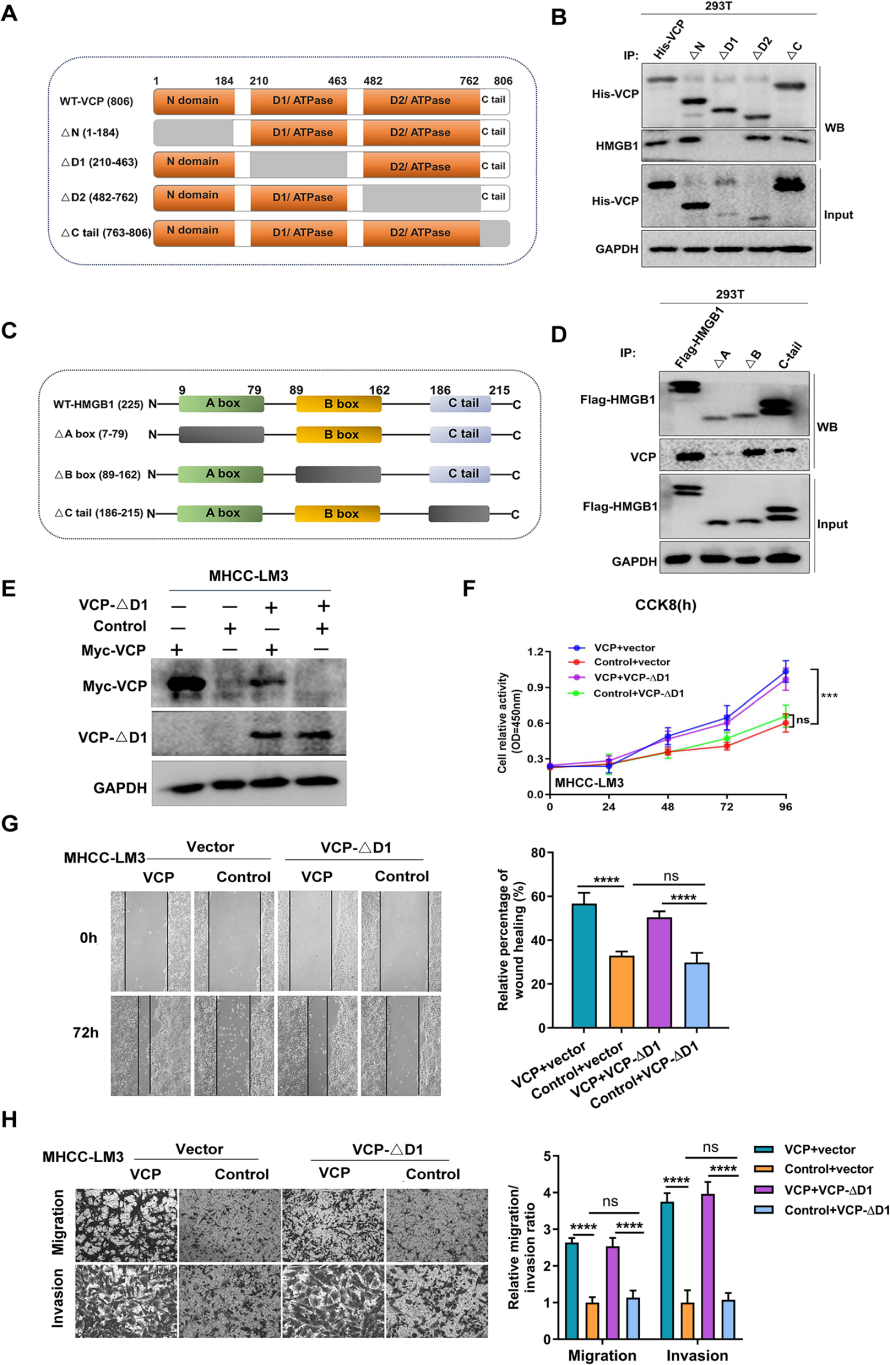
The D1 domain is the key region for VCP exhibiting biological function in HCC. **A** The scheme of VCP mutants. The WT VCP including 806 amino acids was divided into four domains (△N, △D1, △D2, and △C tail). The gray area indicated the deleted region. **B** 293T cells were transfected with His-tagged full-length VCP and its mutants with deletion of various regions. Cell lysates were immunoprecipitated with anti-His antibody. And western blot was used to detect the expression of His probe and HMGB1. **C** The scheme of HMGB1. The full length of HMGB1 included 215 amino acids and was divided into three regions including A box, B box, and C-tail. The box with gray color indicates the deleted domain of HMGB1. **D** 293 T cells were transfected with WT HMGB1 and its various mutants with the expression of the Flag probe. Cell lysates were collected for immunoprecipitation with anti-Flag antibody. Then the expression of Flag probe and VCP were determined by western blot. **E** The protein expression of WT VCP and D1 mutant in MHCC-LM3 cells. **F** The ability of cell proliferation was measured by CCK8 assay in MHCC-LM3 cells with transfection of WT VCP and D1 mutant, separately. **G** The movement capacity of MHCC-LM3 cells with transfection of WT VCP or D1 mutant was evaluated by wound-healing assay. **H** Transwell system indicates the cell's ability of invasion and migration in various groups. The representative images and the corresponding statistical results were shown. WT: wild type. All ***P < 0.001, ****P < 0.0001, and *ns* no significance

Next, we aimed to elucidate the crucial protein domains that are responsible for the interaction between VCP and HMGB1. Based on their protein structures [5, 8], truncated mutation plasmids of VCP (wild type (WT), ΔN, ΔD1, ΔD2, and ΔC tail) and HMGB1 (WT, ΔA box, ΔB box, and ΔC tail) were constructed as indicated (Fig. 6A, C). Then, the series of VCP or HMGB1 mutant plasmids were transfected into 293T cells and CoIP was performed separately. The result revealed that the deletion of the D1 domain in VCP and A box in HMGB1 significantly disrupted the interaction (Fig. 6B, D). The influence of the D1 domain on VCP in promoting HCC progression was also investigated. To this end, exogenous VCP-ΔD1 was transfected into MHCC-LM3 cells, and the transfection efficacy was determined (Fig. 6E). The CCK8 assay showed that the cell proliferative ability was not increased in cells treated only with VCP-ΔD1, but it was enhanced significantly within the existing full-length VCP (Fig. 6F). Similarly, the D1 mutant did not promote cell migration and invasion (Fig. 6G, H). These data support that the D1 region plays a critical role in VCP exhibiting pro-HCC activities.

### VCP increases HMGB1 protein stability

**Fig. 7:**
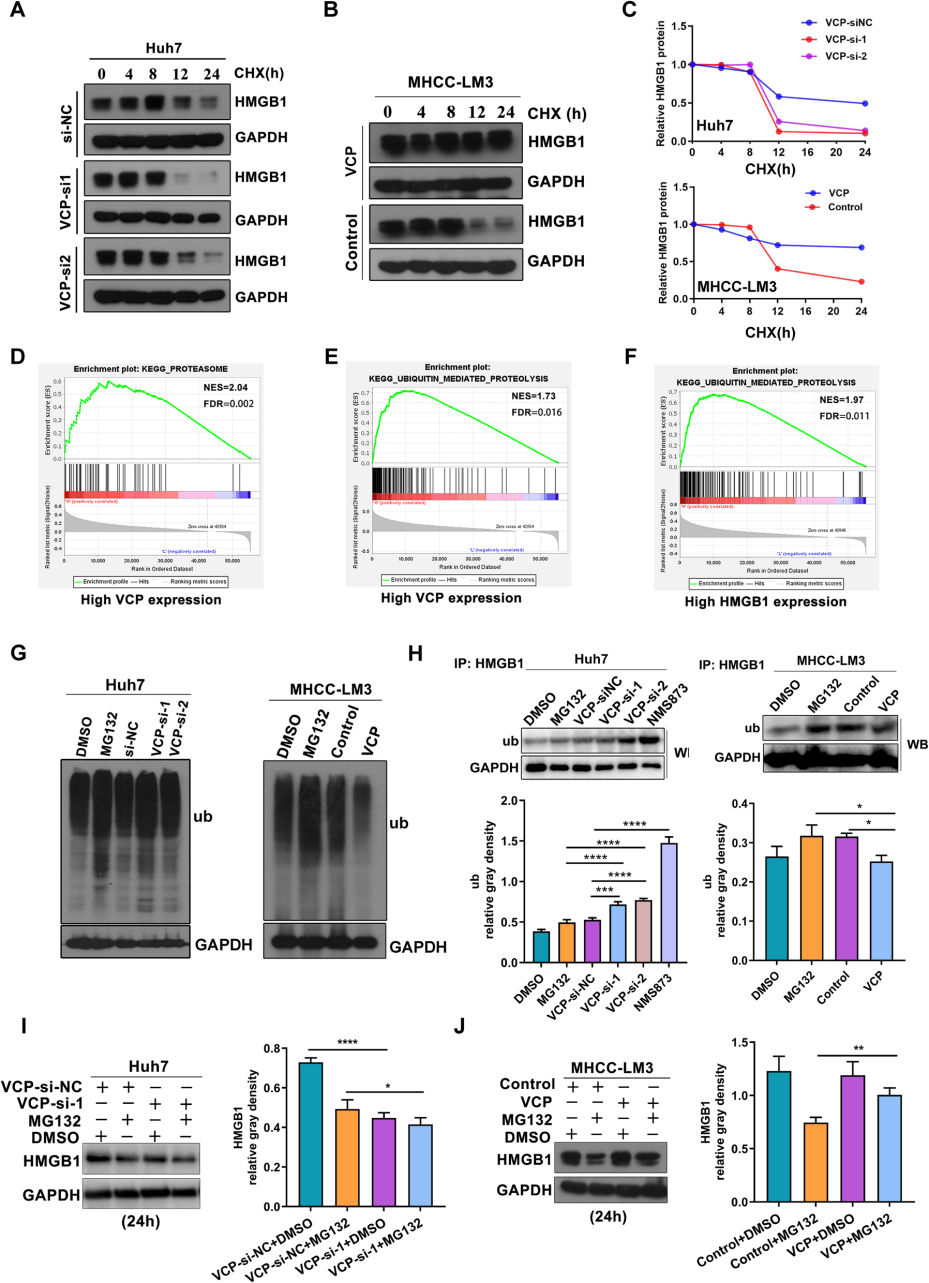
VCP increases the protein stability of HMGB1. **A**–**C** Western blot was performed to evaluate the half-life of HMGB1 protein in HCC cells. All groups were treated with cycloheximide (CHX, 50 μg/mL), a classic protein synthesis inhibitor, from 0 to 24 h. **D**–**F** The GSEA analysis indicated the enriched pathways in HCC patients of high VCP and HMGB1 expressing groups from TCGA database, respectively. The median value of VCP and HMGB1 transcriptional levels in the HCC cohort was regarded as the cut-off. **G** The total cellular levels of ubiquitylated proteins in HCC cells was determined by western blot. Both Huh7 and MHCC-LM3 cells were treated with MG132 (20 μM, 6 h), which is a general proteasome inhibitor. **H** The ubiquitylated HMGB1 protein was detected by western blot after HMGB1 immunoprecipitation in HCC cells. Cells were treated with MG132 (20 μM, 6 h) or the VCP inhibitor NMS873 (10 μM, 6 h). **I**, **J** The Huh7 cells with VCP depletion and MHCC-LM3 cells with ectopic VCP overexpression were incubated with MG132 (20 μM) for 24 h. The whole-cell lysates were collected and separated by SDS-PAGE. Proteins were detected by indicated antibodies. All *P < 0.05, **P < 0.01, and ****P < 0.0001

At first, the HMGB1 expression in HCC cells and immortalized hepatocytes was examined by western blot, showing the elevated expression of HMGB1 in tumor cells (Additional file 6: Fig. S3). Moreover, the results found that the increased VCP expression significantly up-regulated the protein level of HMGB1 instead of the transcriptional expression (Additional file 7: Fig. S4). Then, the impact of VCP on the half-life of HMGB1 was investigated. Under normal circumstances, HMGB1 is rapidly degraded within 24 h. In contrast, the half-life of HMGB1 protein was dramatically prolonged in cells with VCP overexpression, and this phenomenon was reversed in cells with VCP knockdown (Fig. 7A–C).

Next, GSEA in the HCC cohort from TCGA database showed that the process of proteasome and ubiquitin-mediated proteolysis was enriched in the group with high VCP expression. Simultaneously, the up-regulated signal of ubiquitin-mediated proteolysis was also detected in the group with elevated HMGB1 expression (Fig. 7D–F). The overall ubiquitination level of HCC cells was examined after incubation with MG132 (an inhibitor of proteasome) or other indicated treatment. The results demonstrated a distinct increase in cellular ubiquitylated proteins in cells with VCP inhibition. The corresponding decreased ubiquitylated level in cells overexpressing VCP was observed (Fig. 7G). Concurrently, the ubiquitylated level of HMGB1 in HCC cells displayed the same tendency following different VCP expression levels (Fig. 7H). Furthermore, we detected the overall expression of HMGB1 in HCC cells after treating with MG132 (20 μM, 24 h), it was noticed that the protein expressed level of HMGB1 was enhanced in cells overexpressing VCP, and largely reduced when silencing VCP expression (Fig. 7I, J). These findings show that HMGB1 is a substrate of VCP-dependent proteasomal degradation.

### HMGB1 is essential for VCP-mediated PI3K/AKT/mTOR pathway activation in HCC

**Fig. 8:**
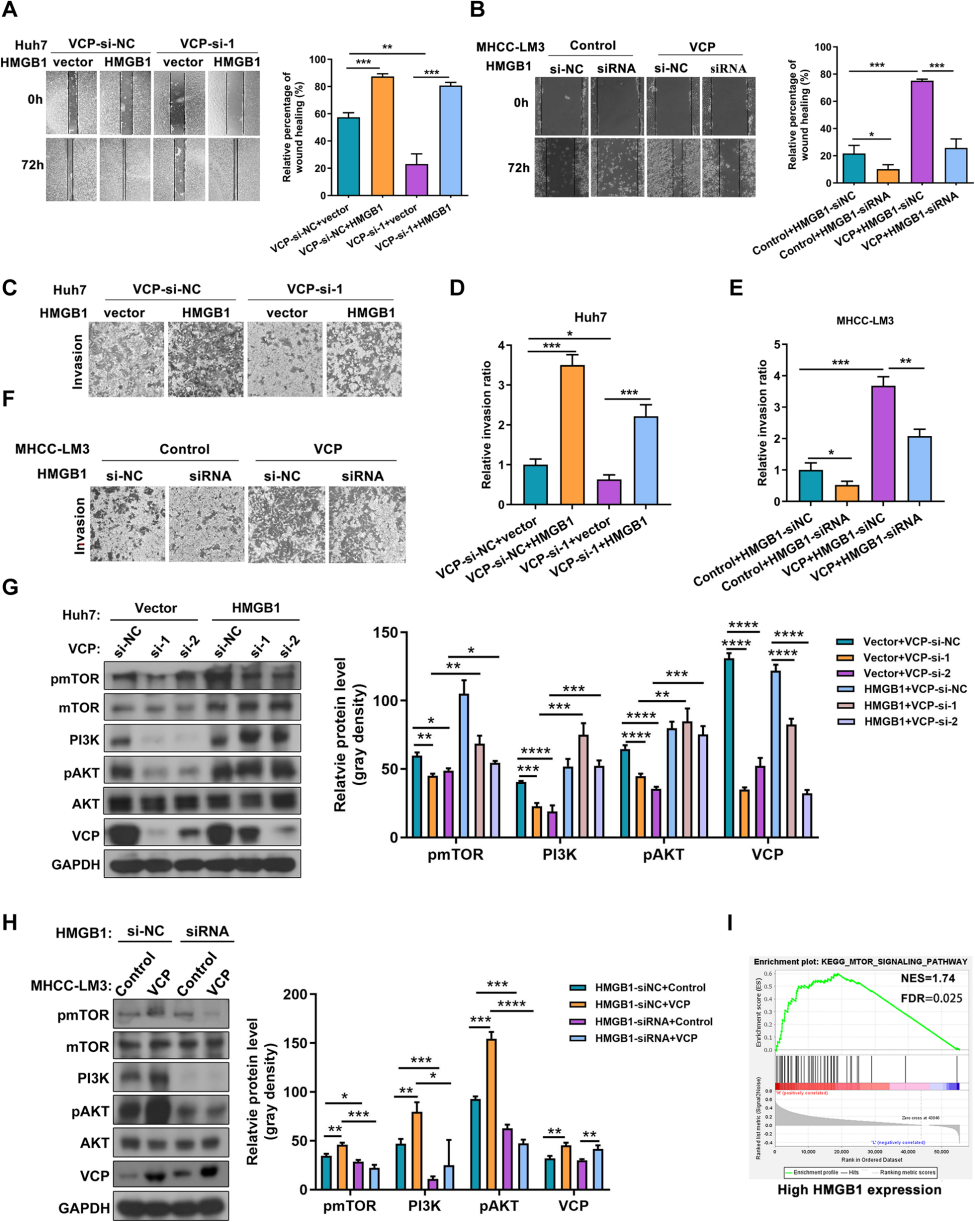
HMGB1 is critical for VCP to activate P13K/AKT/mTOR pathway in HCC cells. **A**, **B** The wound-healing assay was operated to test the moveable ability of Huh7 and MHCC-LM3 cells with indicated treatment. **C**–**F** The transwell system was used to evaluate the cell’s invasive capacity. **G**, **H** The influence of HMGB1 expression on VCP triggering the PI3K/AKT/mTOR pathway was assessed by western blot. The representative results and quantitative analyzed data were shown. **I** The GSEA analysis showed that the mTOR pathway was enriched in HCC patients with high expression of HMGB1 from TCGA database. The median value of HMGB1 transcriptional expression in the HCC cohort was adopted to classify patients into high and low HMGB1 expressed groups. All *P < 0.05, **P < 0.01, ***P < 0.001, and ****P < 0.0001

To elucidate the role of HMGB1 in oncogenic activities mediated by VCP in HCC progression. Firstly, the efficacy of siRNA targeting HMGB1 was verified in HCC cells, among which HMGB1-si-1 was used for the following experiments, referred to here as HMGB1-siRNA (Additional file 8: Fig. S5). In vitro data showed that the enhanced invasive ability of HCC cells induced by VCP was dramatically counteracted by HMGB1 inhibition. In contrast, when overexpressing HMGB1 in HCC cells with VCP silencing, cell invasion was rescued (Fig. 8A–F). Simultaneously, the strengthened EMT mediated by elevated VCP expression was impeded by HMGB1 knockdown (Additional file 9: Fig. S6). The findings indicate that HMGB1 is critical for the biological function of VCP in promoting HCC. Furthermore, the impact of HMGB1 on VCP in activating the mTOR signaling was evaluated. Functionally, the increased levels of phosphorylated AKT, mTOR, and PI3K by VCP overexpression were significantly attenuated by knockdown of HMGB1. In contrast, the exogenous expression of HMGB1 enhanced the phosphorylated levels of biomarkers within the mTOR pathway in cells with VCP knockdown (Fig. 8G, H). GSEA in the HCC cohort from TCGA database also displayed the enrichment of the mTOR pathway in the high-VCP expression group (Fig. 8I). These results reveal the essential function of HMGB1 for VCP promoting the PI3K/AKT/mTOR pathway activation in HCC.

## Discussion

Currently, HCC ranks as the sixth most common malignant tumor and is thought of as an important medical problem worldwide [4, 27]. It is known that the initiation and progression of HCC are associated with multiple complex processes such as sustained inflammatory damage, cirrhosis formation, hepatocyte necrosis, and regeneration [28, 29]. In the past decades, an increasing number of alterations in genetic and epigenetic levels of functional molecules involved in the tumorigenesis of tumors have been gradually identified [1, 4]. However, the precise molecular mechanisms in HCC remain unclear. The identification of more valuable biomarkers correlated to the progression and prognosis of HCC is still warranted.

VCP is reported to involve in multiple disease states including neurodegenerative disorders and several tumor types such as squamous cell carcinoma and prostate cancer [7, 30]. In a clinical retrospective study performed by Yamamoto et al. in 330 patients with gastric cancer, they found that the expression of VCP was elevated in 233 patients (71.3%), and patients with higher VCP expression experienced larger tumor size. In addition, the expressed VCP level is an independent indicator of poor OS and DFS [11]. Similarly, the correlation between increased VCP expression and poor prognosis has also been observed in NSCLC, esophageal carcinoma, and colorectal tumors [10, 31, 32]. Consistently, in the current study, we found a remarkable increase in VCP expression in tumor tissues compared to adjacent non-tumorous samples. A poorer OS and DFS also occurred in HCC patients with higher VCP levels. Moreover, the elevated VCP expression promoted tumor growth in nude mice models bearing subcutaneous HCC. This implies that VCP expression has a potential prognostic value in HCC progression.

Next, the biological behavior of VCP in HCC cells was investigated using various cell models. The findings revealed that overexpression of VCP significantly enhanced the proliferative ability of HCC cells. Correspondingly, the apoptotic capacity was attenuated. In addition, the ability of migration and invasion in HCC cells was markedly facilitated after ectopic overexpressing VCP via activating the EMT and the PI3K/AKT/mTOR pathway. Similarly, another study showed that the inhibition of VCP apparently suppressed the proliferation and migration of NSCLC cells. In contrast, apoptosis was accelerated, and the cell cycle was arrested in the G0/G1 phase. They further found that VCP directly regulates the protein expression of p53 and NF-κB [33]. Consistent results were also observed in an HCC study, and data demonstrated that the initiation and progression of HCC in vivo were substantially suppressed by VCP depletion. Moreover, miR-129-5p was demonstrated to negatively regulate the expression of VCP, resulting in hindering the progression of HCC [9]. Taken together, these data suggest that VCP is involved in the progression of several cancer types including HCC via multiple mechanisms.

To the current knowledge, VCP is an evolutionarily conserved ATPase molecule that structurally includes two critical regions of the D1 and D2 domains. VCP could interact with a variety of functional substrates to participate in various cellular functions, among which the protein degradation mediated by ubiquitin–proteasome is the most widely researched currently [7, 34]. A study reported that HBX protein, which is a multifunctional protein involved in the transactivation of NF-κB in HBV-related diseases, was validated to interact with VCP both in vitro and in vivo. Furthermore, their interaction visibly activated the NF-κB pathway in HCC cells [35]. Recently, with the increasing recognition of the importance of VCP in the carcinogenesis and progression of tumors, the potential regulatory mechanisms and substrates of VCP have been gradually identified. However, the profile and nature of the cellular substrates remain poorly defined. In this study, a total of 79 VCP-interacting candidates were screened and diverse important molecular functions, including ubiquitin-specific protease, biological processes such as cell–cell adhesion, and signal pathways such as apoptosis and protein ubiquitination were discovered. The findings help us to better understand the profile of VCP substrates and their physiological functions and give us new insight into identifying novel biomarkers associated with HCC progression and prognosis.

HMGB1 is a chromosomal nonhistone protein and has location-specific functional roles within the nucleus as a DNA chaperone, within the cytoplasm to sustain autophagy, and outside cells to interact with multiple receptors [16, 36–38]. A series of studies demonstrated its oncogenic activity in the pathogenesis of HCC through various regulatory mechanisms such as activating advanced glycation end products (RAGE), mTOR pathway, as well as interacting with functional markers like HBX protein [18, 22, 39]. In the current study, the interaction between HMGB1 and VCP was verified in HCC cells. Moreover, the D1 domain in VCP and the A box in HMGB1 were demonstrated to be the critical areas for their correlation. We also illustrated the crucial role of the D1 domain in the process of VCP promoting the proliferation, migration, and invasion in HCC. Besides, the data revealed that knockdown of HMGB1 attenuated the migratory and invasive capacity and blocked the activation of the PI3K/AKT/mTOR pathway triggered by VCP overexpression. In brief, the findings suggest that HMGB1 is a member of the VCP substrate family and plays a critical role in VCP promoting HCC progression. However, more in vitro and in vivo studies regarding the in-depth regulation of VCP triggering the mTOR pathway via interacting with HMGB1 are still warranted in the future.

Hitherto, several studies reported that VCP regulated the process of protein degradation mediated by the ubiquitin–proteasome. However, unlike other involved shuttle proteins, which simply bind to the ubiquitin chains and act as components of the proteasome, VCP interacts with ubiquitinated substrates with the help of cofactors such as Ufd1-Npl4 or p47. As a result, it structurally remodeled or unfolded the target protein using the energy of ATP hydrolysis. This helped the ubiquitin-modified protein to either recycle assisted by deubiquitination enzymes (DUBs) or accelerate degradation by proteasomes. A study reported that VCP suppression perturbs cellular ubiquitylation with the enhancement of ubiquitylated levels of different protein subsets, such as K-6 linked ubiquitylation that is relied on the ubiquitin E3 ligase HUWE1[40]. Our data showed that the HMGB1 stability was distinctly increased after VCP overexpression in HCC cells, and this phenomenon could be reversed when VCP was silenced. The findings indicated that the degradation of HMGB1 mediated by the ubiquitin–proteasome process was largely decreased by up-regulated VCP expression. Further in-depth studies involved in this process, such as the specific ubiquitin chain, cofactor, and enzymes mediating protein degradation or recycling, are required to elucidate these mechanisms in HCC.

In conclusion, we report that the expression of VCP is significantly elevated in HCC and is associated with disease progression and poor outcomes, suggesting that VCP is a promising prognostic factor in HCC patients. Our data demonstrate that VCP functions as an oncogenic gene in the HCC progression by interacting with HMGB1 to activate the PI3K/AKT/mTOR pathway. The newly identified interaction between VCP and HMGB1 may represent a valuable therapeutic target in HCC patients for precise intervention and improvement of survival outcomes.

## Supplementary Information

**Additional file 1: Figure S1.** The increased expression of VCP gene in various cancer types from TCGA database. **A** The mRNA expression of VCP is significantly elevated in various cancers including HCC. **B** The up-regulated mRNA expressed level of VCP in HCC than normal tissues was observed in different races. All **P < 0.01, and ***P < 0.001.

**Additional file 2: Table S1. **The correlation of VCP expression and clinicopathologic features in HCC patients in GSE14520 dataset from GEO database.

**Additional file 3: Table S2.** The VCP-interacting proteins that were identified by co-immunoprecipitation combined with the mass spectrometry technology (CoIP/MS).

**Additional file 4: Figure S2. **The enrichment analysis of GO and pathway for potential proteins that interacted with VCP identified by Co-IP/MS. **A**–**C **Top 20 GO terms of biological process, cell component, and molecular function, respectively, with all P values < 0.05. **D**, **E **Top 20 enriched pathways through KEGG and DisGeNET database, respectively. BP: biological process. CC: cell component. MF: molecular function. GO: gene ontology. KEGG: Kyoto Encyclopedia of Genes and Genomes.

**Additional file 5: Table S3.** The biological process of 79 VCP-interacting proteins that obtained after overlapping the candidate proteins identified in CoIP/MS and TCGA database.

**Additional file 6: Figure S3. **The protein expression of HMGB1 in various HCC cells. All **P < 0.01, and ***P < 0.001.

**Additional file 7: Figure S4. **VCP directly regulated the protein expressed level of HMGB1 instead of transcriptional expression. **A**, **B** The transcriptional expression of HMGB1 in Huh7 cells treated with VCP-siRNA and MHCC-LM3 cells transfected by ectopic VCP. **C**, **D **The protein expression of HMGB1 was decreased in Huh7 cells when VCP expression was inhibited via siRNA or small molecule NMS873 (10 μΜ, 12 h). **E**, **F** the protein expression of HMGB1 was upregulated in MHCC-LM3 cells with ectopic overexpressing VCP. All *P < 0.05, **P < 0.01, ****P < 0.0001, and ns: no significance.

**Additional file 8: Figure S5. **The efficacy verification of HMGB1-siRNA in HCC cells. **A** The protein expression of HMGB1 was determined by western blot. **B **the relative gray density of HMGB1 protein was analyzed by Image J software. ***P < 0.001.

**Additional file 9: Figure S6. **HMGB1 is critical for VCP enhancing epithelial mesenchymal transformation (EMT) in HCC cells. **A**, **B **Huh7 cells were transfected by VCP-siRNA and exogenous HMGB1. Meanwhile, MHCC-LM3 cells were treated with exogenous VCP and HMGB1-siRNA. Western blot was operated to determine the protein expression of biomarkers relevant to EMT, and the gray density was further analyzed. All *P < 0.05, **P < 0.01, ***P < 0.001 , and ****P < 0.0001.

## Data Availability

All data generated or analyzed during this study are included in this published article and its supplementary information files.

## Abbreviations

HCC: Hepatocellular carcinoma
HBX: Hepatitis B virus X protein
HMGB1: High-mobility group box 1
VCP: Valosin-containing protein
AAA: ATPases associated with various cellular activities
ER: Endoplasmic reticulum
TCGA: The Cancer Genome Atlas Program
GEO: Gene expression omnibus
DEMs: Differentially expressed mRNAs
GSEA: Gene set enrichment analysis
WT: Wild-type
GO: Gene ontology
SDS-PAGE: Sodium dodecyl sulfate–polyacrylamide gel electrophoresis
PVDF: Polyvinylidene difluoride
FBS: Fetal bovine serum
FDR: False discovery rate
OS: Overall survival
DFS: Disease-free survival
TNM: Tumor-node-metastasis
EMT: Epithelial-mesenchymal transition
NES: Normalized enrichment score
NSCLC: Non-small cell lung carcinoma
RAGE: Activating advanced glycation end products
TLRs: Toll-like receptors
