# Supplementary material for: VCP interaction with HMGB1 promotes hepatocellular carcinoma progression by activating the PI3K/AKT/mTOR pathway
Source: J Transl Med. 2022 May 13;20:212. doi: 10.1186/s12967-022-03416-5 (PMC9102726; doi:10.1186/s12967-022-03416-5)
Supplement: Supplementary file 1 — Additional file 1: Figure S1. The increased expression of VCP gene in various cancer types from TCGA database. A The mRNA expression of VCP is significantly elevated in various cancers including HCC. B The up-regulated mRNA expressed level of VCP in HCC than normal tissues was observed in different races. All **P < 0.01, and ***P < 0.001. [file 12967_2022_3416_MOESM1_ESM.pptx]

## Slide 1
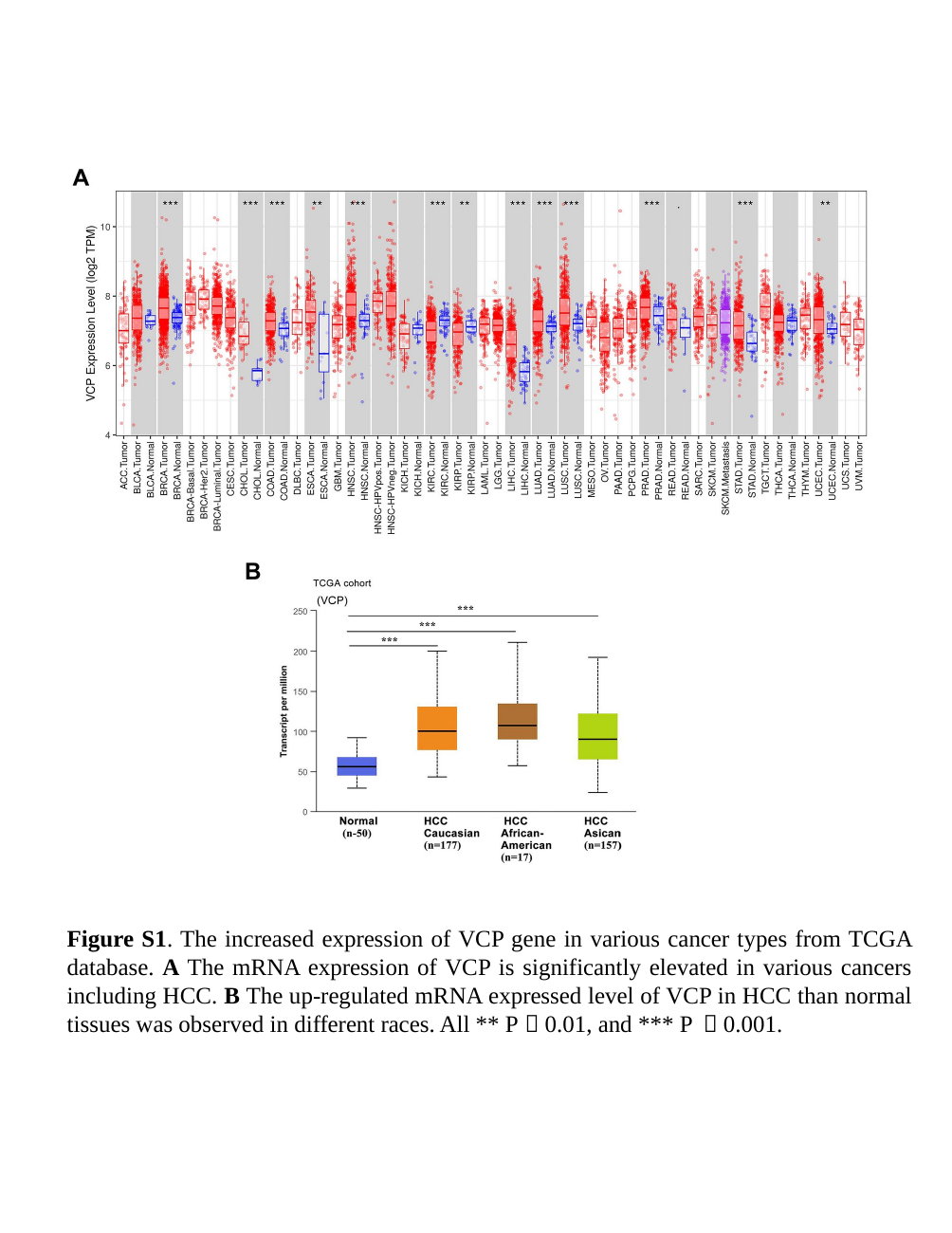

Figure S1. The increased expression of VCP gene in various cancer types from TCGA database. A The mRNA expression of VCP is significantly elevated in various cancers including HCC. B The up-regulated mRNA expressed level of VCP in HCC than normal tissues was observed in different races. All ** P＜0.01, and *** P ＜0.001.
