# Supplementary material for: VCP interaction with HMGB1 promotes hepatocellular carcinoma progression by activating the PI3K/AKT/mTOR pathway
Source: J Transl Med. 2022 May 13;20:212. doi: 10.1186/s12967-022-03416-5 (PMC9102726; doi:10.1186/s12967-022-03416-5)
Supplement: Supplementary file 2 — Additional file 2: Table S1. The correlation of VCP expression and clinicopathologic features in HCC patients in GSE14520 dataset from GEO database. [file 12967_2022_3416_MOESM2_ESM.docx]

**Table S1.** The correlation of VCP expression and clinicopathologic features in HCC patients in GSE14520 dataset from GEO database.

| **Variables** |  | **VCP expressed level** ^a^ | | | | | | |
| --- | --- | --- | --- | --- | --- | --- | --- | --- |
|  |  | **All cases** |  | **Low expression** |  | **High expression** |  | **P-value** ^b^ |
| **Age (years ^c^)** |  |  |  |  |  |  |  | 0.100 |
| ＜50 |  | 78 |  | 44 (56.4%) |  | 34 (43.6%) |  |  |
| ≥50 |  | 85 |  | 37 (43.5%) |  | 48 (56.5%) |  |  |
| **Gender** |  |  |  |  |  |  |  | 0.082 |
| Male |  | 144 |  | 68 (47.2%) |  | 76 (52.8%) |  |  |
| Female |  | 19 |  | 13 (68.4%) |  | 6 (31.6%) |  |  |
| **AFP (ng/ml)** |  |  |  |  |  |  |  | 0.677 |
| ＜300 |  | 89 |  | 46 (51.7%) |  | 43 (48.3%) |  |  |
| ≥300 |  | 71 |  | 33 (46.5%) |  | 38 (53.5%) |  |  |
| **ALT (U/L)** |  |  |  |  |  |  |  | 0.172 |
| ＜50 |  | 96 |  | 52 (54.2%) |  | 44 (45.8%) |  |  |
| ≥50 |  | 67 |  | 29 (43.3%) |  | 38 (56.7%) |  |  |
| **cirrhosis** |  |  |  |  |  |  |  | 0.984 |
| Yes |  | 153 |  | 76 (49.7%) |  | 77 (50.3%) |  |  |
| No |  | 10 |  | 5 (50%） |  | 5 (50%) |  |  |
| **tumor size (cm)** |  |  |  |  |  |  |  | 0.193 |
| ＜5 |  | 102 |  | 55 (53.9%) |  | 47 (46.1%) |  |  |
| ≥5 |  | 60 |  | 26 (43.3%) |  | 34 (56.7%) |  |  |
| **tumor multiplicity** |  |  |  |  |  |  |  | 0.595 |
| Single |  | 128 |  | 65 (50.8%) |  | 33 (49.2%) |  |  |
| Muliple |  | 35 |  | 16 (45.7%) |  | 19 (54.3%) |  |  |
| **TNM stage** |  |  |  |  |  |  |  | 0.03* |
| I-II |  | 115 |  | 65 (56.5%) |  | 50 (43.5%) |  |  |
| III-IV |  | 34 |  | 12 (35.5%) |  | 22 (64.7) |  |  |
| **BCLC** |  |  |  |  |  |  |  | 0.171 |
| early stage |  | 111 |  | 61 (55.5%) |  | 50 (45%) |  |  |
| advanced stage |  | 38 |  | 16 (42.1%) |  | 22 (57.9%) |  |  |

^a^ Patients whose VCP expression levels were in the upper one-third were regarded as the high VCP expressing group, and those whose expression levels were in the lower one-third were distributed to the low expression group. ^b^ Chi-square test. ^c^ median age. AFP: alpha-fetoprotein. TNM: tumor-node-metastasis. BCLC: Barcelona-Clinic Liver Cancer staging system.
