# Supplementary material for: VCP interaction with HMGB1 promotes hepatocellular carcinoma progression by activating the PI3K/AKT/mTOR pathway
Source: J Transl Med. 2022 May 13;20:212. doi: 10.1186/s12967-022-03416-5 (PMC9102726; doi:10.1186/s12967-022-03416-5)
Supplement: Supplementary file 3 — Additional file 3: Table S2. The VCP-interacting proteins that were identified by co-immunoprecipitation combined with the mass spectrometry technology (CoIP/MS). [file 12967_2022_3416_MOESM3_ESM.docx]

**Table S2**. The VCP-interacting proteins that were identified by co-immunoprecipitation combined with the mass spectrometry technology (CoIP/MS).

| **Master Protein Accession** | **Gene** | **Coverage [%]** | **Peptides** | **Unique Peptides** | **MW [kDa]** |
| --- | --- | --- | --- | --- | --- |
| P55072 | TERA | 67 | 41 | 41 | 89.3 |
| P49327 | FAS | 22 | 36 | 36 | 273.3 |
| P11413 | G6PD | 76 | 31 | 31 | 59.2 |
| P13645 | K1C10 | 63 | 34 | 31 | 58.8 |
| P04264 | K2C1 | 54 | 36 | 31 | 66 |
| P35527 | K1C9 | 65 | 27 | 27 | 62 |
| P05787 | K2C8 | 62 | 30 | 26 | 53.7 |
| P07237 | PDIA1 | 54 | 25 | 25 | 57.1 |
| P35908 | K22E | 66 | 31 | 24 | 65.4 |
| Q9Y223 | GLCNE | 42 | 24 | 24 | 79.2 |
| P22314 | UBA1 | 30 | 23 | 23 | 117.8 |
| P00558 | PGK1 | 67 | 23 | 23 | 44.6 |
| P07355 | ANXA2 | 62 | 23 | 23 | 38.6 |
| P23141 | EST1 | 45 | 22 | 22 | 62.5 |
| P11142 | HSP7C | 44 | 26 | 21 | 70.9 |
| P11021 | BIP | 40 | 24 | 21 | 72.3 |
| P43490 | NAMPT | 51 | 20 | 20 | 55.5 |
| O60701 | UGDH | 59 | 19 | 19 | 55 |
| P60842 | IF4A1 | 53 | 19 | 19 | 46.1 |
| P30101 | PDIA3 | 50 | 18 | 18 | 56.7 |
| O60218 | AK1BA | 72 | 18 | 18 | 36 |
| P00338 | LDHA | 60 | 19 | 18 | 36.7 |
| P02786 | TFR1 | 28 | 17 | 17 | 84.8 |
| P00352 | AL1A1 | 52 | 19 | 17 | 54.8 |
| P11216 | PYGB | 31 | 19 | 16 | 96.6 |
| Q9UGI8 | TES | 47 | 16 | 16 | 48 |
| P04406 | G3P | 65 | 16 | 16 | 36 |
| P02545 | LMNA | 30 | 16 | 16 | 74.1 |
| P49368 | TCPG | 34 | 15 | 15 | 60.5 |
| P50990 | TCPQ | 35 | 15 | 15 | 59.6 |
| P06733 | ENOA | 58 | 18 | 15 | 47.1 |
| P60174 | TPIS | 71 | 15 | 15 | 30.8 |
| P68104 | EF1A1 | 51 | 14 | 14 | 50.1 |
| P07195 | LDHB | 49 | 15 | 14 | 36.6 |
| P08758 | ANXA5 | 52 | 14 | 14 | 35.9 |
| P06576 | ATPB | 43 | 14 | 14 | 56.5 |
| P37837 | TALDO | 43 | 14 | 14 | 37.5 |
| P07099 | HYEP | 33 | 13 | 13 | 52.9 |
| P15121 | ALDR | 57 | 13 | 13 | 35.8 |
| Q16881 | TRXR1 | 33 | 12 | 12 | 70.9 |
| O75083 | WDR1 | 28 | 12 | 12 | 66.2 |
| P0DMV9 | HS71B | 47 | 22 | 12 | 70 |
| P26641 | EF1G | 35 | 12 | 12 | 50.1 |
| P40926 | MDHM | 41 | 11 | 11 | 35.5 |
| P62701 | RS4X | 39 | 11 | 11 | 29.6 |
| P23396 | RS3 | 51 | 11 | 11 | 26.7 |
| P00390 | GSHR | 36 | 10 | 10 | 56.2 |
| P12956 | XRCC6 | 22 | 10 | 10 | 69.8 |
| P23528 | COF1 | 66 | 10 | 10 | 18.5 |
| P11940 | PABP1 | 18 | 10 | 10 | 70.6 |
| P14923 | PLAK | 16 | 10 | 10 | 81.7 |
| P35900 | K1C20 | 32 | 11 | 10 | 48.5 |
| P31948 | STIP1 | 22 | 10 | 10 | 62.6 |
| P16615 | AT2A2 | 11 | 9 | 9 | 114.7 |
| P13647 | K2C5 | 28 | 18 | 9 | 62.3 |
| P63104 | 1433Z | 57 | 13 | 9 | 27.7 |
| Q06830 | PRDX1 | 55 | 10 | 9 | 22.1 |
| P25705 | ATPA | 21 | 9 | 9 | 59.7 |
| P23526 | SAHH | 19 | 9 | 9 | 47.7 |
| P39023 | RL3 | 30 | 9 | 9 | 46.1 |
| P04843 | RPN1 | 16 | 8 | 8 | 68.5 |
| Q9Y617 | SERC | 25 | 8 | 8 | 40.4 |
| P50454 | SERPH | 30 | 8 | 8 | 46.4 |
| P60709 | ACTB | 66 | 19 | 8 | 41.7 |
| Q9BQE3 | TBA1C | 67 | 21 | 8 | 49.9 |
| O75874 | IDHC | 29 | 9 | 8 | 46.6 |
| P50991 | TCPD | 21 | 8 | 8 | 57.9 |
| P08195 | 4F2 | 19 | 8 | 8 | 68 |
| Q15233 | NONO | 19 | 9 | 8 | 54.2 |
| P15311 | EZRI | 23 | 14 | 7 | 69.4 |
| Q02878 | RL6 | 27 | 7 | 7 | 32.7 |
| O00571 | DDX3X | 14 | 8 | 7 | 73.2 |
| P40227 | TCPZ | 18 | 7 | 7 | 58 |
| P02768 | ALBU | 12 | 7 | 7 | 69.3 |
| P05023 | AT1A1 | 9 | 7 | 7 | 112.8 |
| Q04637 | IF4G1 | 6 | 7 | 7 | 175.4 |
| P19474 | RO52 | 18 | 7 | 7 | 54.1 |
| P23284 | PPIB | 33 | 7 | 7 | 23.7 |
| P15559 | NQO1 | 28 | 7 | 7 | 30.8 |
| P04792 | HSPB1 | 45 | 7 | 7 | 22.8 |
| Q02790 | FKBP4 | 20 | 7 | 7 | 51.8 |
| Q14974 | IMB1 | 8 | 6 | 6 | 97.1 |
| P31947 | 1433S | 36 | 9 | 6 | 27.8 |
| P62937 | PPIA | 50 | 6 | 6 | 18 |
| P29692 | EF1D | 35 | 6 | 6 | 31.1 |
| P05388 | RLA0 | 27 | 6 | 6 | 34.3 |
| P18124 | RL7 | 29 | 6 | 6 | 29.2 |
| P35659 | DEK | 18 | 6 | 6 | 42.6 |
| P26599 | PTBP1 | 16 | 6 | 6 | 57.2 |
| P17844 | DDX5 | 12 | 7 | 6 | 69.1 |
| P07339 | CATD | 17 | 6 | 6 | 44.5 |
| P22626 | ROA2 | 19 | 6 | 6 | 37.4 |
| Q9UHD8 | 9-Sep | 13 | 6 | 6 | 65.4 |
| O75390 | CISY | 13 | 6 | 6 | 51.7 |
| Q14204 | DYHC1 | 1 | 6 | 6 | 532.1 |
| P05091 | ALDH2 | 16 | 7 | 5 | 56.3 |
| P13489 | RINI | 16 | 5 | 5 | 49.9 |
| P30153 | 2AAA | 10 | 5 | 5 | 65.3 |
| P68366 | TBA4A | 54 | 18 | 5 | 49.9 |
| P02538 | K2C6A | 26 | 16 | 5 | 60 |
| P16152 | CBR1 | 40 | 7 | 5 | 30.4 |
| Q9UJ72 | ANX10 | 22 | 5 | 5 | 37.3 |
| P62826 | RAN | 29 | 5 | 5 | 24.4 |
| Q5QNW6 | H2B2F | 40 | 5 | 5 | 13.9 |
| P61247 | RS3A | 22 | 5 | 5 | 29.9 |
| P62805 | H4 | 50 | 5 | 5 | 11.4 |
| P37802 | TAGL2 | 27 | 5 | 5 | 22.4 |
| P51149 | RAB7A | 31 | 5 | 5 | 23.5 |
| Q92598 | HS105 | 9 | 6 | 5 | 96.8 |
| P34932 | HSP74 | 10 | 6 | 5 | 94.3 |
| P63241 | IF5A1 | 42 | 5 | 5 | 16.8 |
| P15170 | ERF3A | 13 | 5 | 5 | 55.7 |
| Q16555 | DPYL2 | 17 | 5 | 5 | 62.3 |
| Q01518 | CAP1 | 14 | 5 | 5 | 51.9 |
| O00429 | DNM1L | 7 | 5 | 5 | 81.8 |
| P61221 | ABCE1 | 11 | 5 | 5 | 67.3 |
| Q8WX93 | PALLD | 4 | 5 | 5 | 150.5 |
| Q15645 | PCH2 | 12 | 5 | 5 | 48.5 |
| P53396 | ACLY | 6 | 5 | 5 | 120.8 |
| P32119 | PRDX2 | 22 | 5 | 5 | 21.9 |
| P16435 | NCPR | 9 | 5 | 5 | 76.6 |
| O60506 | HNRPQ | 7 | 5 | 5 | 69.6 |
| Q01082 | SPTB2 | 3 | 5 | 5 | 274.4 |
| P41250 | GARS | 8 | 5 | 5 | 83.1 |
| P41091 | IF2G | 12 | 4 | 4 | 51.1 |
| P07437 | TBB5 | 70 | 21 | 4 | 49.6 |
| P02533 | K1C14 | 24 | 11 | 4 | 51.5 |
| P05141 | ADT2 | 36 | 10 | 4 | 32.8 |
| P61981 | 1433G | 32 | 8 | 4 | 28.3 |
| Q15366 | PCBP2 | 25 | 7 | 4 | 38.6 |
| O95373 | IPO7 | 5 | 4 | 4 | 119.4 |
| P62241 | RS8 | 24 | 4 | 4 | 24.2 |
| Q92688 | AN32B | 19 | 4 | 4 | 28.8 |
| Q9NQC3 | RTN4 | 6 | 4 | 4 | 129.9 |
| P13804 | ETFA | 19 | 4 | 4 | 35.1 |
| Q8NBS9 | TXND5 | 13 | 4 | 4 | 47.6 |
| P31689 | DNJA1 | 13 | 4 | 4 | 44.8 |
| Q9Y678 | COPG1 | 6 | 4 | 4 | 97.7 |
| P40121 | CAPG | 13 | 4 | 4 | 38.5 |
| Q14444 | CAPR1 | 8 | 4 | 4 | 78.3 |
| P18621 | RL17 | 25 | 4 | 4 | 21.4 |
| P61019 | RAB2A | 27 | 4 | 4 | 23.5 |
| O00299 | CLIC1 | 27 | 4 | 4 | 26.9 |
| P30040 | ERP29 | 18 | 4 | 4 | 29 |
| P40925 | MDHC | 16 | 4 | 4 | 36.4 |
| Q14847 | LASP1 | 18 | 4 | 4 | 29.7 |
| Q9Y3F4 | STRAP | 17 | 4 | 4 | 38.4 |
| P40429 | RL13A | 18 | 4 | 4 | 23.6 |
| P62269 | RS18 | 26 | 4 | 4 | 17.7 |
| P35232 | PHB | 16 | 4 | 4 | 29.8 |
| P51114 | FXR1 | 9 | 4 | 4 | 69.7 |
| Q15046 | SYK | 8 | 4 | 4 | 68 |
| Q9Y265 | RUVB1 | 11 | 4 | 4 | 50.2 |
| P62851 | RS25 | 24 | 4 | 4 | 13.7 |
| Q9NUQ9 | FA49B | 18 | 4 | 4 | 36.7 |
| P53621 | COPA | 3 | 4 | 4 | 138.3 |
| P62280 | RS11 | 23 | 4 | 4 | 18.4 |
| P54578 | UBP14 | 12 | 4 | 4 | 56 |
| P09429 | HMGB1 | 30 | 5 | 4 | 24.9 |
| P12004 | PCNA | 15 | 4 | 4 | 28.8 |
| P27635 | RL10 | 24 | 4 | 4 | 24.6 |
| Q14152 | EIF3A | 2 | 4 | 4 | 166.5 |
| P32969 | RL9 | 36 | 4 | 4 | 21.9 |
| Q92597 | NDRG1 | 13 | 3 | 3 | 42.8 |
| O43399 | TPD54 | 17 | 3 | 3 | 22.2 |
| P68371 | TBB4B | 67 | 20 | 3 | 49.8 |
| P52895 | AK1C2 | 63 | 16 | 3 | 36.7 |
| Q13509 | TBB3 | 40 | 14 | 3 | 50.4 |
| P15531 | NDKA | 47 | 6 | 3 | 17.1 |
| Q15365 | PCBP1 | 22 | 6 | 3 | 37.5 |
| Q15185 | TEBP | 25 | 3 | 3 | 18.7 |
| P13797 | PLST | 10 | 5 | 3 | 70.8 |
| Q07021 | C1QBP | 12 | 3 | 3 | 31.3 |
| P06748 | NPM | 14 | 3 | 3 | 32.6 |
| P62633 | CNBP | 24 | 3 | 3 | 19.5 |
| P24534 | EF1B | 19 | 3 | 3 | 24.7 |
| P26373 | RL13 | 15 | 3 | 3 | 24.2 |
| Q07020 | RL18 | 17 | 3 | 3 | 21.6 |
| P48735 | IDHP | 11 | 4 | 3 | 50.9 |
| P40939 | ECHA | 5 | 3 | 3 | 82.9 |
| O43708 | MAAI | 17 | 3 | 3 | 24.2 |
| P30050 | RL12 | 25 | 3 | 3 | 17.8 |
| Q53H82 | LACB2 | 16 | 3 | 3 | 32.8 |
| P62136 | PP1A | 14 | 3 | 3 | 37.5 |
| P62917 | RL8 | 13 | 3 | 3 | 28 |
| P09651 | ROA1 | 10 | 3 | 3 | 38.7 |
| P29966 | MARCS | 16 | 3 | 3 | 31.5 |
| Q9NTK5 | OLA1 | 9 | 3 | 3 | 44.7 |
| Q16181 | 7-Sep | 10 | 4 | 3 | 50.6 |
| Q13283 | G3BP1 | 8 | 3 | 3 | 52.1 |
| P30048 | PRDX3 | 13 | 3 | 3 | 27.7 |
| P49736 | MCM2 | 4 | 3 | 3 | 101.8 |
| Q16891 | MIC60 | 5 | 3 | 3 | 83.6 |
| P30740 | ILEU | 9 | 3 | 3 | 42.7 |
| Q96AG4 | LRC59 | 11 | 3 | 3 | 34.9 |
| P62987 | RL40 | 24 | 3 | 3 | 14.7 |
| Q01105 | SET | 13 | 3 | 3 | 33.5 |
| P04632 | CPNS1 | 12 | 3 | 3 | 28.3 |
| Q13263 | TIF1B | 5 | 3 | 3 | 88.5 |
| P24752 | THIL | 8 | 3 | 3 | 45.2 |
| P13010 | XRCC5 | 6 | 3 | 3 | 82.7 |
| B5ME19 | EIFCL | 4 | 3 | 3 | 105.4 |
| Q9Y266 | NUDC | 12 | 3 | 3 | 38.2 |
| P28799 | GRN | 6 | 3 | 3 | 63.5 |
| P30520 | PURA2 | 9 | 3 | 3 | 50.1 |
| Q9Y3Z3 | SAMH1 | 5 | 3 | 3 | 72.2 |
| P05198 | IF2A | 10 | 3 | 3 | 36.1 |
| P84098 | RL19 | 17 | 3 | 3 | 23.5 |
| P00505 | AATM | 9 | 3 | 3 | 47.5 |
| Q04446 | GLGB | 6 | 3 | 3 | 80.4 |
| Q14533 | KRT81 | 5 | 4 | 3 | 54.9 |
| O60716 | CTND1 | 3 | 3 | 3 | 108.1 |
| Q9NSD9 | SYFB | 6 | 3 | 3 | 66.1 |
| P41252 | SYIC | 2 | 3 | 3 | 144.4 |
| O43143 | DHX15 | 6 | 3 | 3 | 90.9 |
| Q9Y4L1 | HYOU1 | 6 | 3 | 3 | 111.3 |
| Q96C19 | EFHD2 | 12 | 3 | 3 | 26.7 |
| P51858 | HDGF | 11 | 2 | 2 | 26.8 |
| P52565 | GDIR1 | 15 | 2 | 2 | 23.2 |
| Q04828 | AK1C1 | 73 | 20 | 2 | 36.8 |
| Q13885 | TBB2A | 49 | 17 | 2 | 49.9 |
| P68133 | ACTS | 36 | 12 | 2 | 42 |
| P08779 | K1C16 | 21 | 9 | 2 | 51.2 |
| P22392 | NDKB | 38 | 5 | 2 | 17.3 |
| P12236 | ADT3 | 26 | 8 | 2 | 32.8 |
| P07951 | TPM2 | 17 | 6 | 2 | 32.8 |
| P00441 | SODC | 17 | 2 | 2 | 15.9 |
| P43487 | RANG | 17 | 2 | 2 | 23.3 |
| Q9UNM6 | PSD13 | 7 | 2 | 2 | 42.9 |
| P62277 | RS13 | 18 | 2 | 2 | 17.2 |
| Q13162 | PRDX4 | 17 | 3 | 2 | 30.5 |
| P18085 | ARF4 | 20 | 3 | 2 | 20.5 |
| P51148 | RAB5C | 11 | 2 | 2 | 23.5 |
| Q99623 | PHB2 | 8 | 2 | 2 | 33.3 |
| O14818 | PSA7 | 12 | 2 | 2 | 27.9 |
| P49755 | TMEDA | 11 | 2 | 2 | 25 |
| O14828 | SCAM3 | 9 | 2 | 2 | 38.3 |
| P46776 | RL27A | 15 | 2 | 2 | 16.6 |
| O60884 | DNJA2 | 5 | 2 | 2 | 45.7 |
| P49748 | ACADV | 4 | 2 | 2 | 70.3 |
| P16401 | H15 | 10 | 2 | 2 | 22.6 |
| P60953 | CDC42 | 15 | 2 | 2 | 21.2 |
| Q9P0L0 | VAPA | 11 | 2 | 2 | 27.9 |
| Q12797 | ASPH | 5 | 2 | 2 | 85.8 |
| P51648 | AL3A2 | 4 | 2 | 2 | 54.8 |
| P25685 | DNJB1 | 6 | 2 | 2 | 38 |
| P11766 | ADHX | 6 | 2 | 2 | 39.7 |
| P28072 | PSB6 | 9 | 2 | 2 | 25.3 |
| O95573 | ACSL3 | 5 | 2 | 2 | 80.4 |
| P53634 | CATC | 5 | 2 | 2 | 51.8 |
| P62491 | RB11A | 10 | 2 | 2 | 24.4 |
| P55786 | PSA | 3 | 2 | 2 | 103.2 |
| Q03135 | CAV1 | 20 | 2 | 2 | 20.5 |
| P50552 | VASP | 7 | 2 | 2 | 39.8 |
| P78417 | GSTO1 | 7 | 2 | 2 | 27.5 |
| O76003 | GLRX3 | 8 | 2 | 2 | 37.4 |
| Q7L273 | KCTD9 | 7 | 2 | 2 | 42.5 |
| Q92820 | GGH | 8 | 2 | 2 | 35.9 |
| Q96I99 | SUCB2 | 5 | 2 | 2 | 46.5 |
| P28066 | PSA5 | 12 | 2 | 2 | 26.4 |
| O00410 | IPO5 | 2 | 2 | 2 | 123.6 |
| O75822 | EIF3J | 9 | 2 | 2 | 29 |
| P25789 | PSA4 | 7 | 2 | 2 | 29.5 |
| Q9HAW8 | UD110 | 5 | 2 | 2 | 59.8 |
| P51572 | BAP31 | 10 | 2 | 2 | 28 |
| Q9ULV4 | COR1C | 5 | 2 | 2 | 53.2 |
| P27708 | PYR1 | 1 | 2 | 2 | 242.8 |
| Q96TA1 | NIBA2 | 4 | 2 | 2 | 84.1 |
| P25205 | MCM3 | 2 | 2 | 2 | 90.9 |
| P06312 | KV401 | 20 | 2 | 2 | 13.4 |
| P00492 | HPRT | 10 | 2 | 2 | 24.6 |
| Q96C36 | P5CR2 | 7 | 2 | 2 | 33.6 |
| P31930 | QCR1 | 5 | 2 | 2 | 52.6 |
| P42766 | RL35 | 15 | 2 | 2 | 14.5 |
| P48163 | MAOX | 3 | 2 | 2 | 64.1 |
| P01892 | HLAA | 7 | 2 | 2 | 40.9 |
| P61160 | ARP2 | 6 | 2 | 2 | 44.7 |
| Q16851 | UGPA | 4 | 2 | 2 | 56.9 |
| P46783 | RS10 | 18 | 2 | 2 | 18.9 |
| P62714 | PP2AB | 10 | 2 | 2 | 35.6 |
| P23588 | IF4B | 4 | 2 | 2 | 69.1 |
| P22695 | QCR2 | 5 | 2 | 2 | 48.4 |
| O43169 | CYB5B | 35 | 2 | 2 | 16.7 |
| P23246 | SFPQ | 5 | 3 | 2 | 76.1 |
| P00325 | ADH1B | 5 | 2 | 2 | 39.8 |
| P60763 | RAC3 | 9 | 2 | 2 | 21.4 |
| P00491 | PNPH | 7 | 2 | 2 | 32.1 |
| Q8TF66 | LRC15 | 3 | 2 | 2 | 64.3 |
| P28482 | MK01 | 5 | 2 | 2 | 41.4 |
| Q16836 | HCDH | 14 | 2 | 2 | 34.3 |
| O95994 | AGR2 | 14 | 2 | 2 | 20 |
| P20290 | BTF3 | 14 | 2 | 2 | 22.2 |
| P54136 | SYRC | 3 | 2 | 2 | 75.3 |
| Q9UBT2 | SAE2 | 4 | 2 | 2 | 71.2 |
| Q99497 | PARK7 | 15 | 2 | 2 | 19.9 |
| Q13404 | UB2V1 | 13 | 2 | 2 | 16.5 |
| P83731 | RL24 | 11 | 2 | 2 | 17.8 |
| Q13347 | EIF3I | 6 | 2 | 2 | 36.5 |
| P13716 | HEM2 | 6 | 2 | 2 | 36.3 |
| Q92945 | FUBP2 | 3 | 2 | 2 | 73.1 |
| P39656 | OST48 | 4 | 2 | 2 | 50.8 |
| Q96D46 | NMD3 | 4 | 2 | 2 | 57.6 |
| Q6DD88 | ATLA3 | 4 | 2 | 2 | 60.5 |
| P31943 | HNRH1 | 6 | 2 | 2 | 49.2 |
| Q9Y262 | EIF3L | 3 | 2 | 2 | 66.7 |
| Q14103 | HNRPD | 6 | 2 | 2 | 38.4 |
| O15254 | ACOX3 | 4 | 2 | 2 | 77.6 |
| O15067 | PUR4 | 2 | 2 | 2 | 144.6 |
| P17812 | PYRG1 | 3 | 2 | 2 | 66.6 |
| Q16698 | DECR | 7 | 2 | 2 | 36 |
| P07737 | PROF1 | 17 | 2 | 2 | 15 |
| P14324 | FPPS | 5 | 2 | 2 | 48.2 |
| Q8WUM4 | PDC6I | 3 | 2 | 2 | 96 |
| P54577 | SYYC | 3 | 2 | 2 | 59.1 |
| P22676 | CALB2 | 8 | 2 | 2 | 31.5 |
| Q02543 | RL18A | 9 | 2 | 2 | 20.7 |
| P50570 | DYN2 | 2 | 2 | 2 | 98 |
| P84243 | H33 | 12 | 2 | 2 | 15.3 |
| Q8NBJ5 | GT251 | 4 | 2 | 2 | 71.6 |
| Q99798 | ACON | 3 | 2 | 2 | 85.4 |
| P49006 | MRP | 14 | 2 | 2 | 19.5 |
| P62081 | RS7 | 10 | 2 | 2 | 22.1 |
| P39748 | FEN1 | 7 | 2 | 2 | 42.6 |
| Q8TCT9 | HM13 | 4 | 2 | 2 | 41.5 |
| P02751 | FINC | 1 | 2 | 2 | 262.5 |
| O00487 | PSDE | 6 | 2 | 2 | 34.6 |
| P05387 | RLA2 | 23 | 2 | 2 | 11.7 |
| Q8NC51 | PAIRB | 3 | 2 | 2 | 44.9 |
| Q96G03 | PGM2 | 3 | 2 | 2 | 68.2 |
