# Supplementary material for: VCP interaction with HMGB1 promotes hepatocellular carcinoma progression by activating the PI3K/AKT/mTOR pathway
Source: J Transl Med. 2022 May 13;20:212. doi: 10.1186/s12967-022-03416-5 (PMC9102726; doi:10.1186/s12967-022-03416-5)
Supplement: Supplementary file 4 — Additional file 4: Figure S2. The enrichment analysis of GO and pathway for potential proteins that interacted with VCP identified by Co-IP/MS. A–C Top 20 GO terms of biological process, cell component, and molecular function, respectively, with all P values < 0.05. D, E Top 20 enriched pathways through KEGG and DisGeNET database, respectively. BP: biological process. CC: cell component. MF: molecular function. GO: gene ontology. KEGG: Kyoto Encyclopedia of Genes and Genomes. [file 12967_2022_3416_MOESM4_ESM.pptx]

## Slide 1
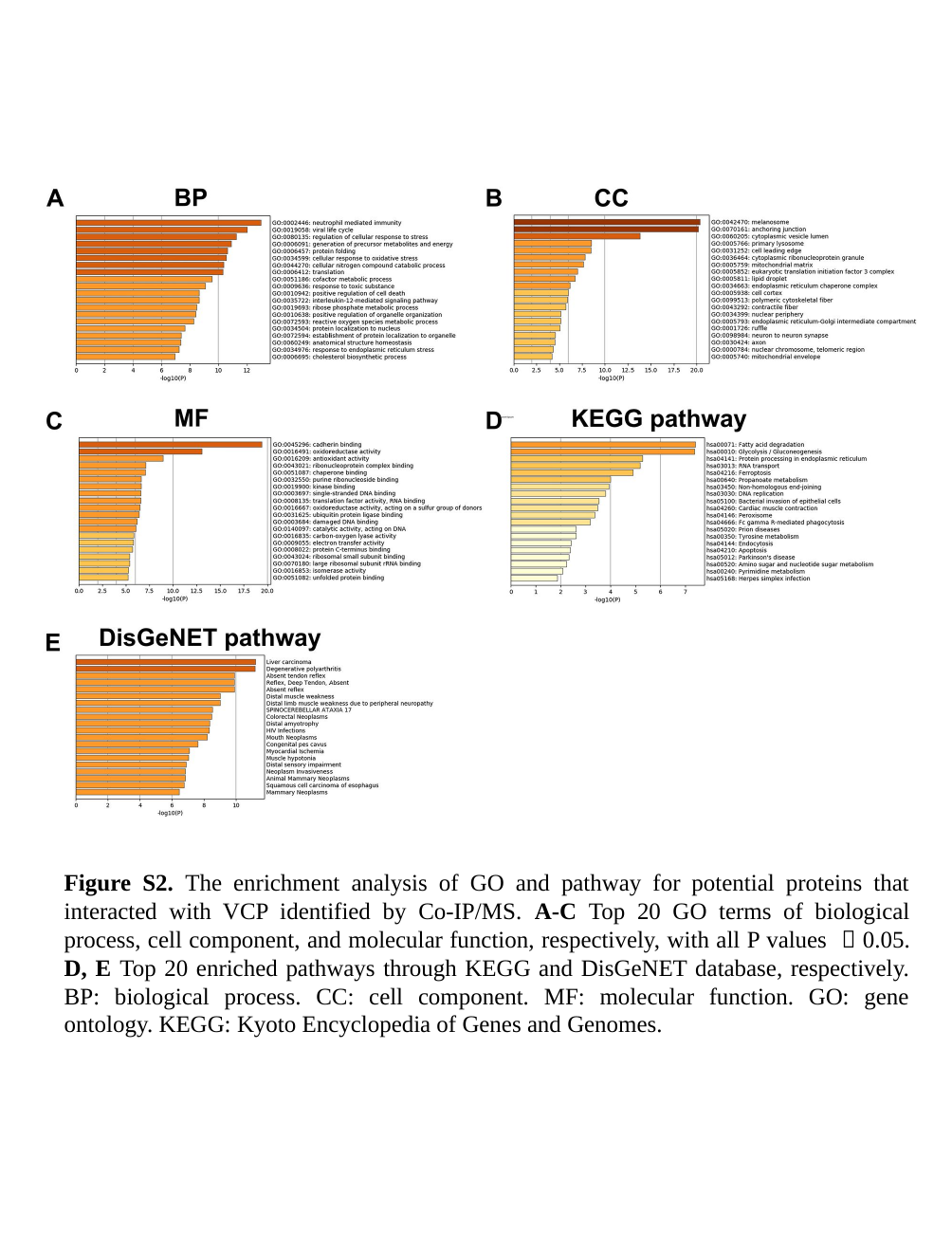

Figure S2. The enrichment analysis of GO and pathway for potential proteins that interacted with VCP identified by Co-IP/MS. A-C Top 20 GO terms of biological process, cell component, and molecular function, respectively, with all P values ＜0.05. D, E Top 20 enriched pathways through KEGG and DisGeNET database, respectively. BP: biological process. CC: cell component. MF: molecular function. GO: gene ontology. KEGG: Kyoto Encyclopedia of Genes and Genomes.
