# Supplementary material for: VCP interaction with HMGB1 promotes hepatocellular carcinoma progression by activating the PI3K/AKT/mTOR pathway
Source: J Transl Med. 2022 May 13;20:212. doi: 10.1186/s12967-022-03416-5 (PMC9102726; doi:10.1186/s12967-022-03416-5)
Supplement: Supplementary file 5 — Additional file 5: Table S3. The biological process of 79 VCP-interacting proteins that obtained after overlapping the candidate proteins identified in CoIP/MS and TCGA database. [file 12967_2022_3416_MOESM5_ESM.docx]

**Table S3**. The biological process of 79 VCP-interacting proteins that obtained after overlapping the candidate proteins identified in CoIP/MS and TCGA database.

| **Category** | **Term** | **Count** | **P-value** | **FDR** |
| --- | --- | --- | --- | --- |
| BP | GO:0098609~cell-cell adhesion | 12 | 3.58E-08 | 5.50E-05 |
| BP | GO:0032508~DNA duplex unwinding | 5 | 4.58E-05 | 0.070491845 |
| BP | GO:0075522~IRES-dependent viral translational initiation | 3 | 4.18E-04 | 0.641477997 |
| BP | GO:0039694~viral RNA genome replication | 3 | 7.13E-04 | 1.090924227 |
| BP | GO:0006888~ER to Golgi vesicle-mediated transport | 6 | 7.90E-04 | 1.208781926 |
| BP | GO:0021762~substantia nigra development | 4 | 0.001413388 | 2.151847626 |
| BP | GO:0016032~viral process | 7 | 0.002332771 | 3.52826072 |
| BP | GO:0000398~mRNA splicing, via spliceosome | 6 | 0.003331974 | 5.003632359 |
| BP | GO:0001731~formation of translation preinitiation complex | 3 | 0.004809135 | 7.14612971 |
| BP | GO:0007264~small GTPase mediated signal transduction | 6 | 0.005143529 | 7.62482651 |
| BP | GO:0006260~DNA replication | 5 | 0.005353908 | 7.9248082 |
| BP | GO:0006310~DNA recombination | 4 | 0.006315479 | 9.284364616 |
| BP | GO:0008380~RNA splicing | 5 | 0.006803887 | 9.967709782 |
| BP | GO:0006397~mRNA processing | 5 | 0.008827419 | 12.74793938 |
| BP | GO:0071475~cellular hyperosmotic salinity response | 2 | 0.009031713 | 13.0241233 |
| BP | GO:0006446~regulation of translational initiation | 3 | 0.011529776 | 16.33578642 |
| BP | GO:0000723~telomere maintenance | 3 | 0.011529776 | 16.33578642 |
| BP | GO:0006695~cholesterol biosynthetic process | 3 | 0.012791126 | 17.9627937 |
| BP | GO:0055114~oxidation-reduction process | 8 | 0.017528767 | 23.81337602 |
| BP | GO:1902177~positive regulation of oxidative stress | 2 | 0.017982919 | 24.35322901 |
| BP | GO:1903377~negative regulation of oxidative stress-induced neuron intrinsic apoptotic signaling pathway | 2 | 0.017982919 | 24.35322901 |
| BP | GO:0045876~positive regulation of sister chromatid cohesion | 2 | 0.017982919 | 24.35322901 |
| BP | GO:0010467~gene expression | 3 | 0.019936727 | 26.63519638 |
| BP | GO:0030036~actin cytoskeleton organization | 4 | 0.021151757 | 28.02166913 |
| BP | GO:0006886~intracellular protein transport | 5 | 0.022203588 | 29.20209382 |
| BP | GO:0032481~positive regulation of type I interferon production | 3 | 0.022339875 | 29.35371095 |
| BP | GO:0006413~translational initiation | 4 | 0.024238431 | 31.43450541 |
| BP | GO:0006749~glutathione metabolic process | 3 | 0.026595585 | 33.93819493 |
| BP | GO:0000055~ribosomal large subunit export from nucleus | 2 | 0.026854324 | 34.20775023 |
| BP | GO:0042256~mature ribosome assembly | 2 | 0.026854324 | 34.20775023 |
| BP | GO:0006606~protein import into nucleus | 3 | 0.02838259 | 35.77904109 |
| BP | GO:0010628~positive regulation of gene expression | 5 | 0.030998321 | 38.38727015 |
| BP | GO:0075713~establishment of integrated proviral latency | 2 | 0.035646632 | 42.7794746 |
| BP | GO:0050687~negative regulation of defense response to virus | 2 | 0.035646632 | 42.7794746 |
| BP | GO:0006302~double-strand break repair | 3 | 0.035990282 | 43.09228247 |
| BP | GO:0002281~macrophage activation involved in immune response | 2 | 0.040013341 | 46.63731444 |
| BP | GO:0071243~cellular response to arsenic-containing substance | 2 | 0.040013341 | 46.63731444 |
| BP | GO:0060316~positive regulation of ryanodine-sensitive calcium-release channel activity | 2 | 0.040013341 | 46.63731444 |
| BP | GO:0048660~regulation of smooth muscle cell proliferation | 2 | 0.040013341 | 46.63731444 |
| BP | GO:0033138~positive regulation of peptidyl-serine phosphorylation | 3 | 0.040056681 | 46.67435461 |
| BP | GO:0003334~keratinocyte development | 2 | 0.044360537 | 50.235264 |
| BP | GO:0071481~cellular response to X-ray | 2 | 0.044360537 | 50.235264 |
| BP | GO:0045087~innate immune response | 6 | 0.045264359 | 50.95424508 |
| BP | GO:0043065~positive regulation of apoptotic process | 5 | 0.047093345 | 52.37957301 |
| BP | GO:0060765~regulation of androgen receptor signaling pathway | 2 | 0.048688305 | 53.59081728 |
| BP | GO:0010524~positive regulation of calcium ion transport into cytosol | 2 | 0.05299673 | 56.72029164 |
| BP | GO:0008154~actin polymerization or depolymerization | 2 | 0.05299673 | 56.72029164 |
| BP | GO:0034329~cell junction assembly | 2 | 0.05299673 | 56.72029164 |
| BP | GO:0007420~brain development | 4 | 0.054999249 | 58.1066532 |
| BP | GO:0060047~heart contraction | 2 | 0.057285899 | 59.6389069 |
| BP | GO:0034975~protein folding in endoplasmic reticulum | 2 | 0.057285899 | 59.6389069 |
| BP | GO:0019079~viral genome replication | 2 | 0.061555896 | 62.36085907 |
| BP | GO:0000380~alternative mRNA splicing, via spliceosome | 2 | 0.061555896 | 62.36085907 |
| BP | GO:0031333~negative regulation of protein complex assembly | 2 | 0.074251697 | 69.47475334 |
| BP | GO:0000082~G1/S transition of mitotic cell cycle | 3 | 0.077965593 | 71.30483543 |
| BP | GO:0007165~signal transduction | 10 | 0.07811488 | 71.37620887 |
| BP | GO:0010880~regulation of release of sequestered calcium ion into cytosol by sarcoplasmic reticulum | 2 | 0.078445847 | 71.53385102 |
| BP | GO:0070987~error-free translesion synthesis | 2 | 0.082621244 | 73.45416189 |
| BP | GO:0010881~regulation of cardiac muscle contraction by regulation of the release of sequestered calcium ion | 2 | 0.082621244 | 73.45416189 |
| BP | GO:0043523~regulation of neuron apoptotic process | 2 | 0.082621244 | 73.45416189 |
| BP | GO:0048025~negative regulation of mRNA splicing, via spliceosome | 2 | 0.090916111 | 76.91518221 |
| BP | GO:0071480~cellular response to gamma radiation | 2 | 0.090916111 | 76.91518221 |
| BP | GO:0090316~positive regulation of intracellular protein transport | 2 | 0.095035745 | 78.47274098 |

BP: biological process. FDR: false discovery rates.
