# Supplementary figures and images for: VCP interaction with HMGB1 promotes hepatocellular carcinoma progression by activating the PI3K/AKT/mTOR pathway
Source: J Transl Med. 2022 May 13;20:212. doi: 10.1186/s12967-022-03416-5 (PMC9102726; doi:10.1186/s12967-022-03416-5)

## Slide 1
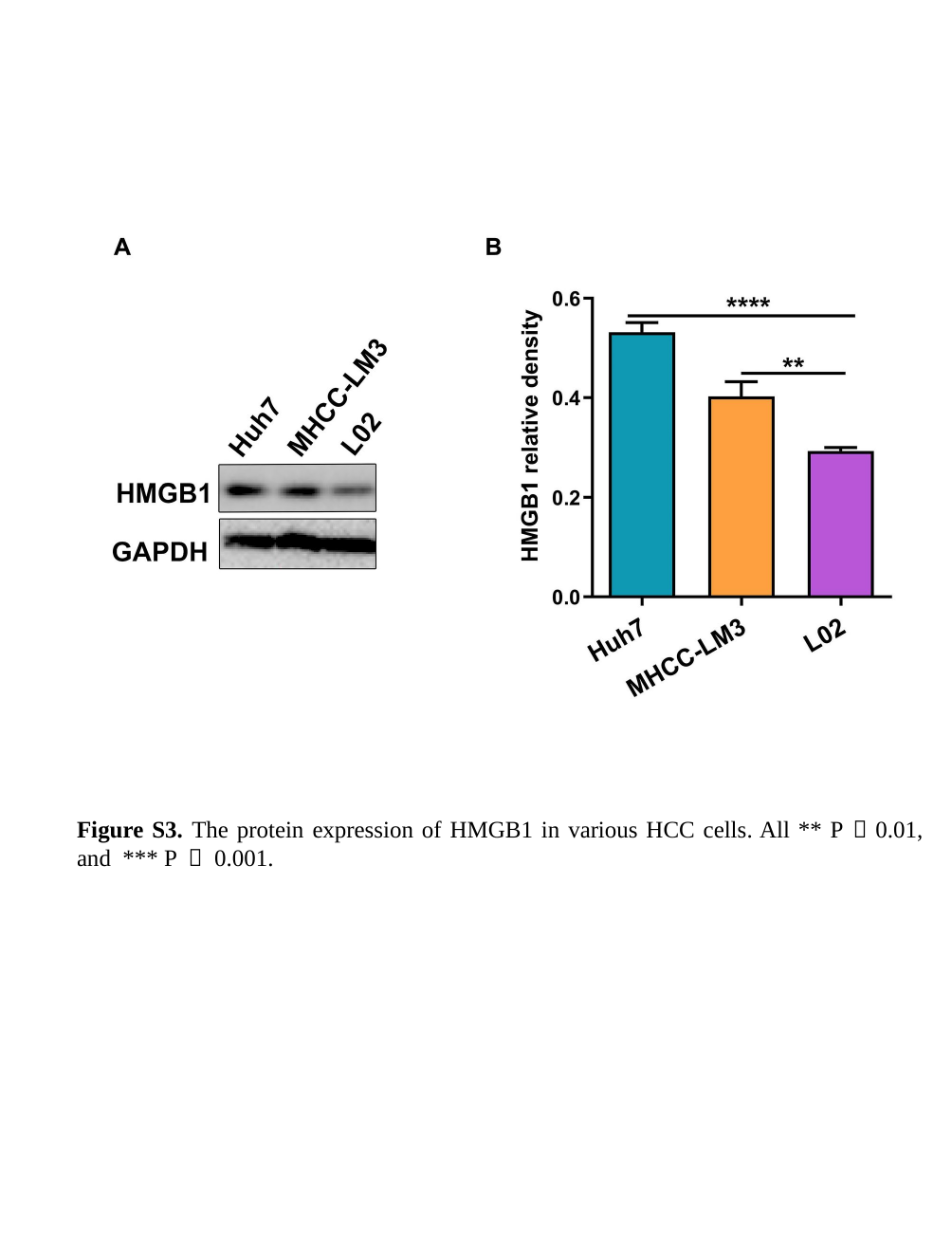

Figure S3. The protein expression of HMGB1 in various HCC cells. All ** P＜0.01, and *** P ＜ 0.001.

Supplement: Supplementary file 6 — Additional file 6: Figure S3. The protein expression of HMGB1 in various HCC cells. All **P < 0.01, and ***P < 0.001. [file 12967_2022_3416_MOESM6_ESM.pptx]
