# Supplementary material for: VCP interaction with HMGB1 promotes hepatocellular carcinoma progression by activating the PI3K/AKT/mTOR pathway
Source: J Transl Med. 2022 May 13;20:212. doi: 10.1186/s12967-022-03416-5 (PMC9102726; doi:10.1186/s12967-022-03416-5)
Supplement: Supplementary file 7 — Additional file 7: Figure S4. VCP directly regulated the protein expressed level of HMGB1 instead of transcriptional expression. A, B The transcriptional expression of HMGB1 in Huh7 cells treated with VCP-siRNA and MHCC-LM3 cells transfected by ectopic VCP. C, D The protein expression of HMGB1 was decreased in Huh7 cells when VCP expression was inhibited via siRNA or small molecule NMS873 (10 μΜ, 12 h). E, F the protein expression of HMGB1 was upregulated in MHCC-LM3 cells with ectopic overexpressing VCP. All *P < 0.05, **P < 0.01, ****P < 0.0001, and ns: no significance. [file 12967_2022_3416_MOESM7_ESM.pptx]

## Slide 1
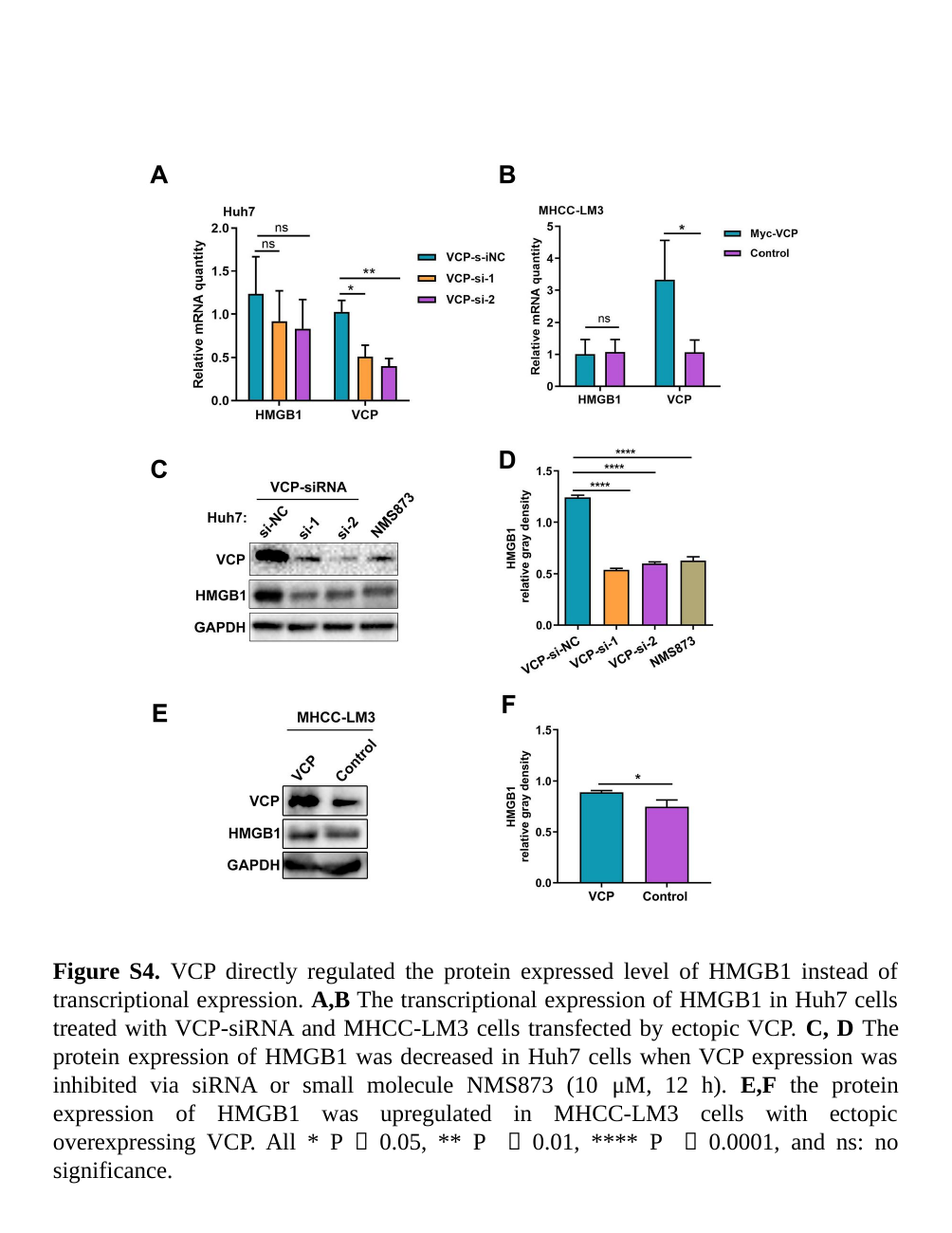

Figure S4. VCP directly regulated the protein expressed level of HMGB1 instead of transcriptional expression. A,B The transcriptional expression of HMGB1 in Huh7 cells treated with VCP-siRNA and MHCC-LM3 cells transfected by ectopic VCP. C, D The protein expression of HMGB1 was decreased in Huh7 cells when VCP expression was inhibited via siRNA or small molecule NMS873 (10 μΜ, 12 h). E,F the protein expression of HMGB1 was upregulated in MHCC-LM3 cells with ectopic overexpressing VCP. All * P＜0.05, ** P ＜0.01, **** P ＜0.0001, and ns: no significance.
