# Supplementary material for: VCP interaction with HMGB1 promotes hepatocellular carcinoma progression by activating the PI3K/AKT/mTOR pathway
Source: J Transl Med. 2022 May 13;20:212. doi: 10.1186/s12967-022-03416-5 (PMC9102726; doi:10.1186/s12967-022-03416-5)
Supplement: Supplementary file 8 — Additional file 8: Figure S5. The efficacy verification of HMGB1-siRNA in HCC cells. A The protein expression of HMGB1 was determined by western blot. B the relative gray density of HMGB1 protein was analyzed by Image J software. ***P < 0.001. [file 12967_2022_3416_MOESM8_ESM.pptx]

## Slide 1
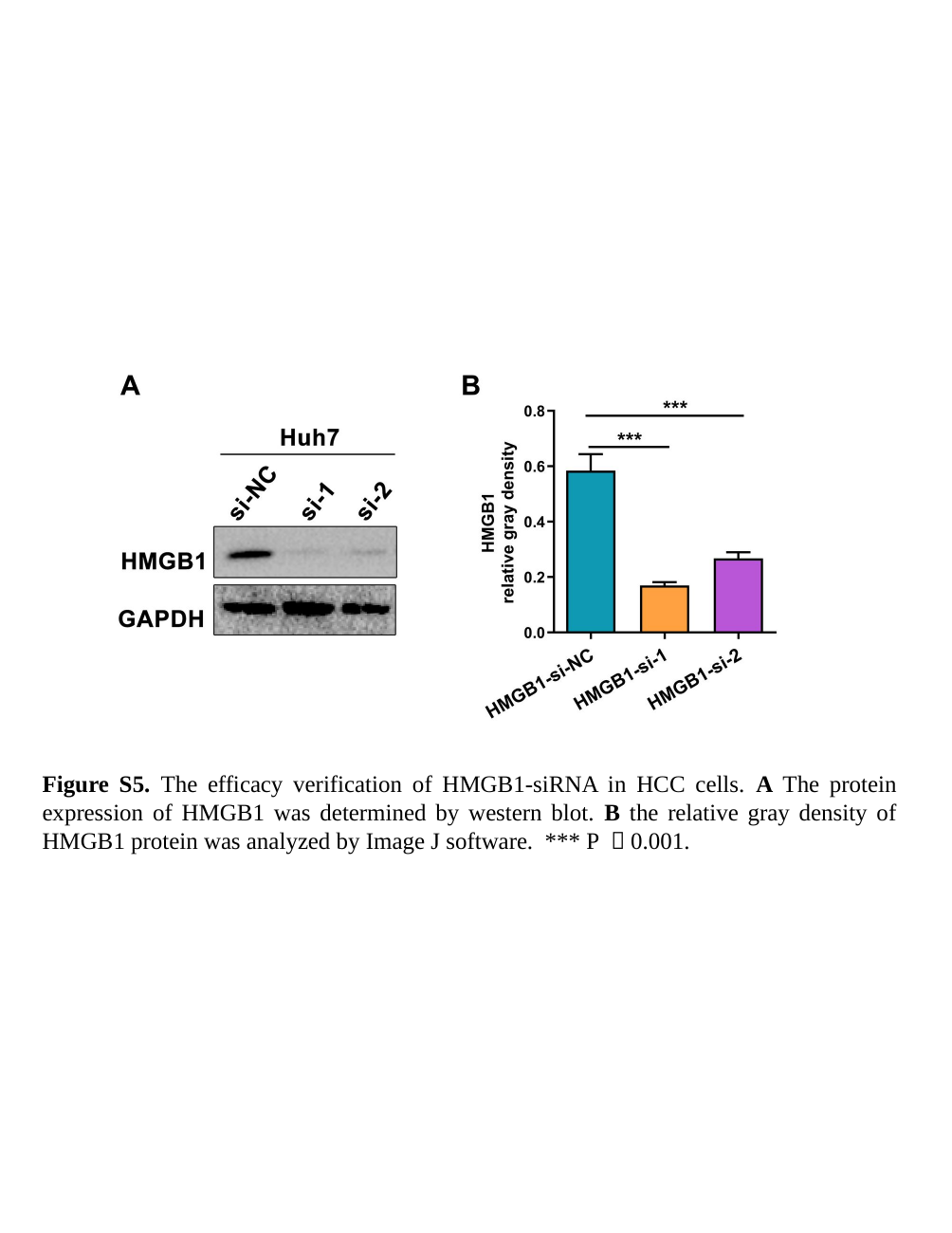

Figure S5. The efficacy verification of HMGB1-siRNA in HCC cells. A The protein expression of HMGB1 was determined by western blot. B the relative gray density of HMGB1 protein was analyzed by Image J software. *** P ＜0.001.
