# Supplementary material for: VCP interaction with HMGB1 promotes hepatocellular carcinoma progression by activating the PI3K/AKT/mTOR pathway
Source: J Transl Med. 2022 May 13;20:212. doi: 10.1186/s12967-022-03416-5 (PMC9102726; doi:10.1186/s12967-022-03416-5)
Supplement: Supplementary file 9 — Additional file 9: Figure S6. HMGB1 is critical for VCP enhancing epithelial mesenchymal transformation (EMT) in HCC cells. A, B Huh7 cells were transfected by VCP-siRNA and exogenous HMGB1. Meanwhile, MHCC-LM3 cells were treated with exogenous VCP and HMGB1-siRNA. Western blot was operated to determine the protein expression of biomarkers relevant to EMT, and the gray density was further analyzed. All *P < 0.05, **P < 0.01, ***P < 0.001 , and ****P < 0.0001. [file 12967_2022_3416_MOESM9_ESM.pptx]

## Slide 1
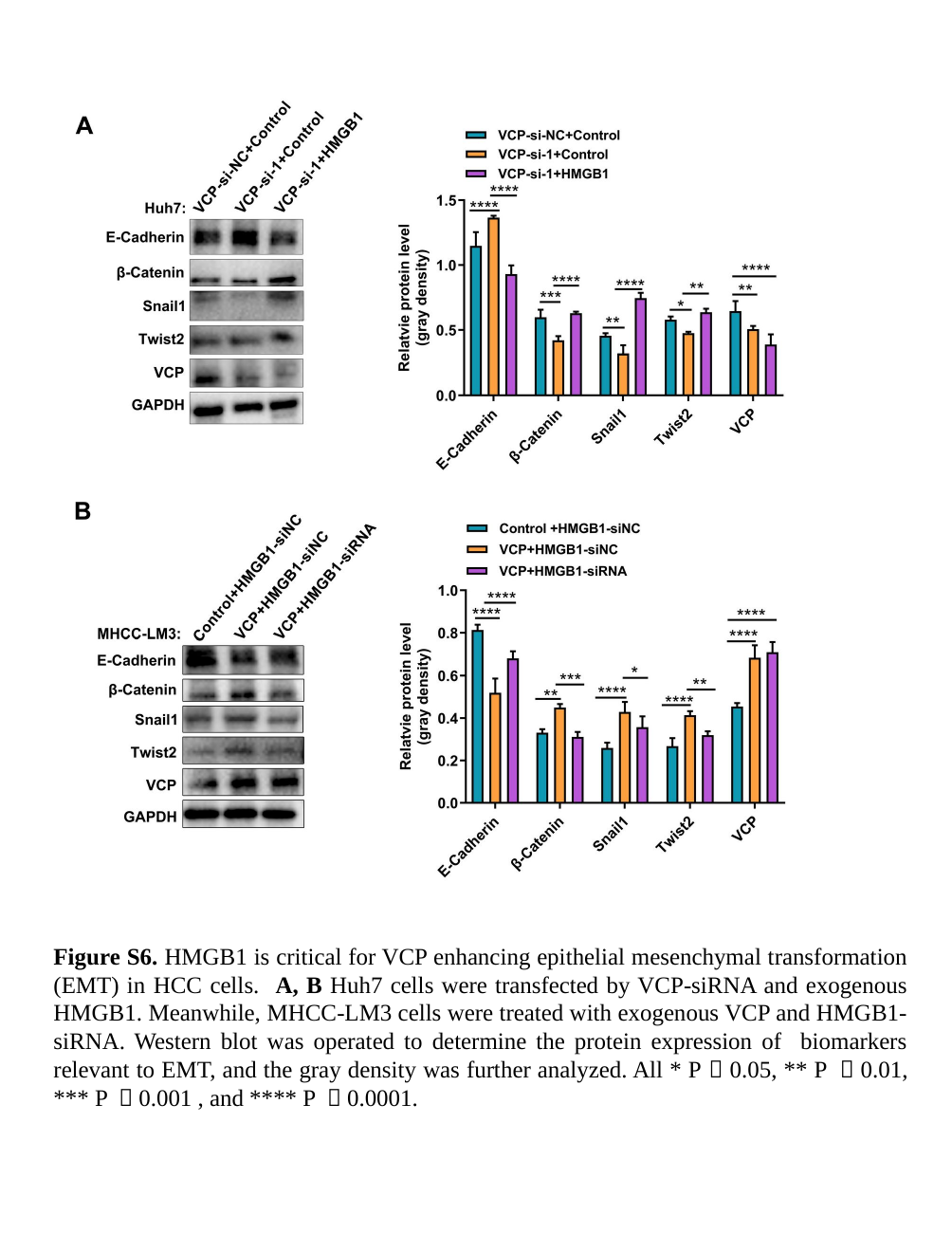

Figure S6. HMGB1 is critical for VCP enhancing epithelial mesenchymal transformation (EMT) in HCC cells. A, B Huh7 cells were transfected by VCP-siRNA and exogenous HMGB1. Meanwhile, MHCC-LM3 cells were treated with exogenous VCP and HMGB1-siRNA. Western blot was operated to determine the protein expression of biomarkers relevant to EMT, and the gray density was further analyzed. All * P＜0.05, ** P ＜0.01, *** P ＜0.001 , and **** P ＜0.0001.
